# *Laminaria japonica* Extract, an Inhibitor of *Clavibater michiganense* Subsp. *Sepedonicum*

**DOI:** 10.1371/journal.pone.0094329

**Published:** 2014-04-08

**Authors:** Jin Cai, Jia Feng, Shulian Xie, Feipeng Wang, Qiufeng Xu

**Affiliations:** School of Life Science, Shanxi University, Taiyuan, People’s Republic of China; University of Nebraska-Lincoln, United States of America

## Abstract

Bacterial ring rot of potato is one of the most serious potato plant and tuber diseases. *Laminaria japonica* extract was investigated for its antimicrobial activity against *Clavibater michiganense* subsp. *sepedonicum* (Spieckermann & Kotthoff) Davis et al., the causative agent of bacterial ring rot of potato. The results showed that the optimum extraction conditions of antimicrobial substances from *L. japonica* were an extraction temperature of 80°C, an extraction time of 12 h, and a solid to liquid ratio of 1∶25. Active compounds of *L. japonica* were isolated by solvent partition, thin layer chromatography (TLC) and column chromatography. All nineteen fractionations had antimicrobial activities against *C. michiganense* subsp. *sepedonicum*, while Fractionation three (Fr.3) had the highest (*P*<0.05) antimicrobial activity. Chemical composition analysis identified a total of 26 components in Fr.3. The main constituents of Fr.3 were alkanes (80.97%), esters (5.24%), acids (4.87%) and alcohols (2.21%). Antimicrobial activity of Fr.3 against *C. michiganense* subsp. *sepedonicum* could be attributed to its ability to damage the cell wall and cell membrane, induce the production of reactive oxygen species (ROS), increase cytosolic Ca^2+^ concentration, inhibit the glycolytic pathway (EMP) and tricarboxylic acid (TCA) cycle, inhibit protein and nucleic acid synthesis, and disrupt the normal cycle of DNA replication. These findings indicate that *L. japonica* extracts have potential for inhibiting *C. michiganense* subsp. *sepedonicum*.

## Introduction

Potato, the third largest global food crop after wheat and rice, is cultivated for its underground storage stems or tubers, which are rich in starch and other nutrients [1]. Bacterial ring rot of potato is one of the most serious potato plant and tuber diseases. It is caused by the gram positive bacterium *Clavibater michiganense* subsp. *sepedonicum* (Spieckermann & Kotthoff) Davis et al., and is a highly infectious disease found in all major potato growing areas [2]. *C. michiganense* subsp. *sepedonicum* may be disseminated by infected tubers, and from them by cutting bags, planters, and knives. *C. michiganense* subsp. *sepedonicum* is difficult to control because it can survive for long periods [3]. Chemical bactericides such as bleach, quaternary ammonia, potassium permanganate, copper sulfate, chlorine dioxide, iodine and phenol–containing compounds are the most commonly used methods for controlling *C. michiganense* subsp. *sepedonicum* [4]–[6]. However, these substances are dangerous to wildlife and humans [7]. In addition, chemical bactericides may persist in the environment for years and are not readily biodegradable [8]–[10]. Plants are exposed to various pathogenic fungi and bacteria [11]. As a countermeasure, plants produce antimicrobial substances that can act as defense mechanisms against phytopathogens [12]–[15].

*Laminaria japonica*, a member of brown algae, is the most important economic seaweed cultured in the Pacific Ocean [16]. In East Asia, it is widely consumed as a marine vegetable [17]. Moreover, *L. japonica* has been used as an herbal medicine in China to treat goiter, scrofula, and dropsy [18], [19]. However, the use of antimicrobial substances from *L. japonica* as potential inhibitors of *C. michiganense* subsp. *Sepedonicum* has not been investigated. The annual production of *L. japonica* in China is about 900,000 t [20], which would provide a good source for the production of antimicrobial substances. The objectives of the present study were (1) to evaluate the antimicrobial activity of *L. japonica* extracts against *C. michiganense* subsp. *sepedonicum*, and to optimize the extraction of *L. japonica* to yield the maximal antimicrobial activity; (2) to separate and determine the major antimicrobial substances from *L. japonica* against *C. michiganense* subsp. *sepedonicum*; and (3) to investigate the possible mechanism of the antimicrobial action. The results indicated that *L. japonica* has a significant potential for inhibiting *C. michiganense* subsp. *sepedonicum*.

## Results

### Optimization of extraction conditions

**Figure 1:**
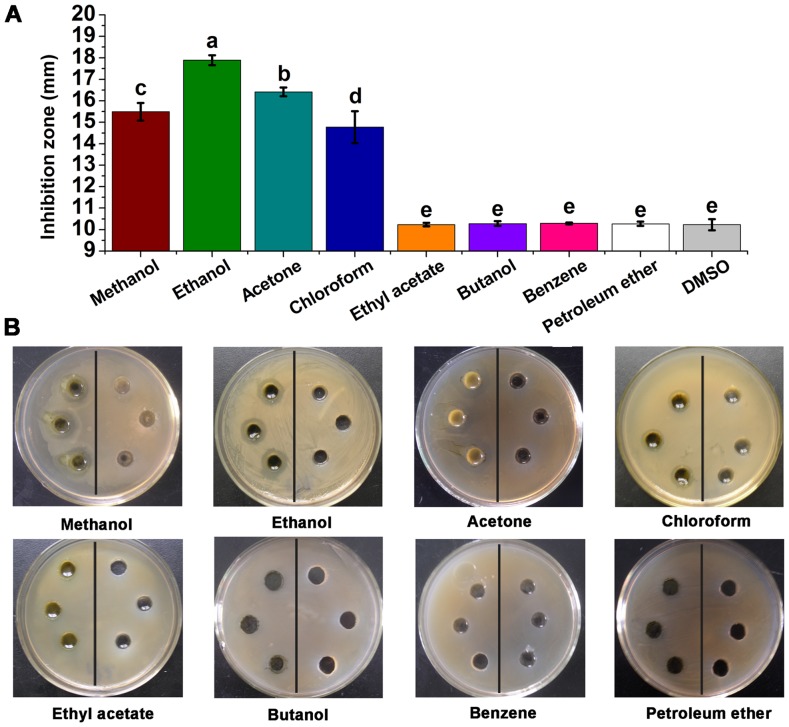
Antimicrobial activities of eight solvent extracts from *L. japonica* against *C. michiganense* subsp. *sepedonicum*. Tests (Figure 1A, B left). DMSO was used as the control (Figure1B right). A negative result is defined as an inhibition zone of 10 mm, while an inhibition zone of greater than 10 mm indicates a positive result. Different letters indicate significant differences (*P*<0.05; ANOVA and Duncan’s multiple range test). Bars represent the mean ± standard deviation. Each experiment was replicated four times.

Extracts obtained by methanol, ethanol, acetone, and chloroform showed obvious antimicrobial activities against *C. michiganense* subsp. *sepedonicum*. However, ethyl acetate, butanol, benzene and petroleum ether extracts showed no antimicrobial activity. The ethanol extracts had the highest inhibition zone value and were significantly (*P*<0.05) different from those obtained with the other solvents (Figure 1).

**Figure 2:**
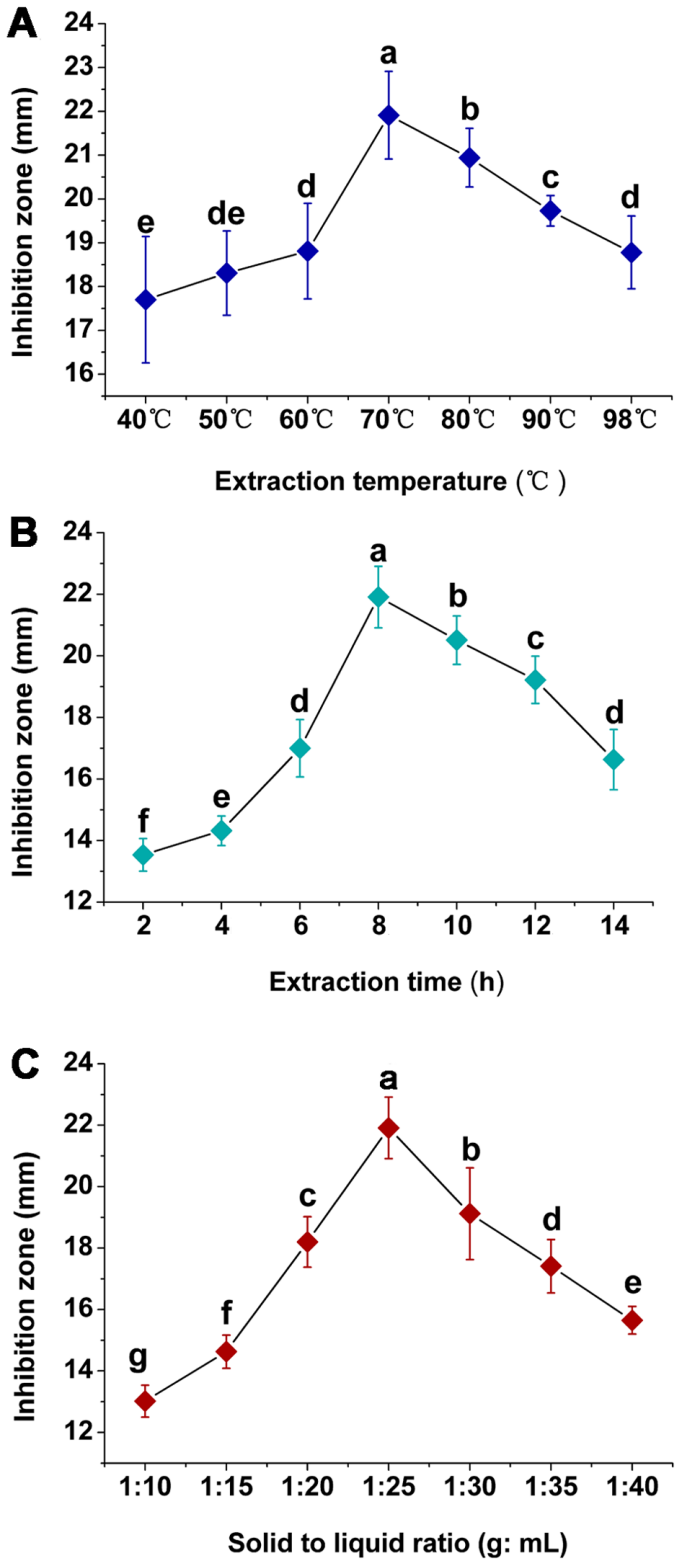
Effects of extraction temperature (A), extraction time (B), and solid to liquid ratio (C). Different letters indicate significant differences (*P*<0.05; ANOVA and Duncan’s multiple range test). Bars represent the mean ± standard error. Each experiment was replicated ten times.

The effects of the extraction temperature, extraction time, and solid to liquid ratio are depicted in Figure 2. As shown in Figure 2A, with an increase in temperature, the diameter of the inhibition zone was increased to a maximum (*P*<0.05) at 70°C. However, further increases in the temperature resulted in a decrease in antimicrobial activity. ANOVA analysis indicated that the extraction temperatures at Level 4 (70°C), Level 5 (80°C), and Level 6 (90°C) were significantly (*P*<0.05) different; thus, they were selected for orthogonal experimental design as shown in Table 1. As shown in Figure 2B, the antimicrobial activity increased with an increase in the extraction time. When the extraction time was 8 h, the diameter of the inhibition zone reached a maximum (*P*<0.05). With longer extraction times, the antimicrobial activity of *L. japonica* decreased. ANOVA analysis indicated that the extraction times at Level 4 (8 h), Level 5 (10 h), and Level 6 (12 h) were significantly (*P*<0.05) different, and thus, they were selected for orthogonal experimental design as shown in Table 1. As shown in Figure 2C, the solid to liquid ratio influenced the antimicrobial activity of *L. japonica.* The diameter of the inhibition zone increased with an increase in the solid to liquid ratio. The maximum (*P*<0.05) extraction yield of antimicrobial substances was achieved at the ratio of 1∶25 while as the amount of solid to liquid increased, the antimicrobial activity decreased. ANOVA analysis indicated that the best (*P*<0.05) solid to liquid ratios were Level 3 (1∶20), Level 4 (1∶25), and Level 5 (1∶30); thus, we selected them for orthogonal experimental design as shown in Table 1.

**Table 1:** Orthogonal design for antimicrobial substance extraction.

Levels	Factors		
	Extraction temperature (A)	Extraction time (B)	Solid to liquid ratio (C)
1	70°C	8 h	1:20 (g: mL)
2	80°C	10 h	1:25 (g: mL)
3	90°C	12 h	1:30 (g: mL)

Orthogonal experimental design in this study was performed to optimize the extraction temperature, extraction time, and the solid to liquid ratio. From experimental results, we inferred that the antimicrobial activity of *L. japonica* was influenced by varying factors at different levels depending on their interactions. A total of 27 groups of tests were carried out according to the orthogonal table L_27_(3^13^) with three factors and three levels as shown in Table 2. The experimental results are shown in Table 2. Factors that influence the antimicrobial activity of *L. japonica* followed the order: A > B, C (Table 2). The individual levels within each factor were ranked as: A: 2>3>1; B: 3>1>2; C: 2>1>3.

**Table 2:** Results obtained under the experimental conditions using L_27_ (3^13^) orthogonal array design.

Test	1	2	3	4	5	6	7	8	9	10	11	12	13	Inhibition zone (mm) a
	A	B	A×B	A×B	C	A×C	A×C	B×C			B×C			
1	1 (70°C)	1 (8 h)	1	1	1 (1:20)	1	1	1	1	1	1	1	1	16.05± 1.23
2	1 (70°C)	1 (8 h)	1	1	2 (1:25)	2	2	2	2	2	2	2	2	16.73± 0.92
3	1 (70°C)	1 (8 h)	1	1	3 (1:30)	3	3	3	3	3	3	3	3	16.32±1.25
4	1 (70°C)	2 (10 h)	2	2	1 (1:20)	1	1	2	2	2	3	3	3	16.58± 1.07
5	1 (70°C)	2 (10 h)	2	2	2 (1:25)	2	2	3	3	3	1	1	1	19.01±1.12
6	1 (70°C)	2 (10 h)	2	2	3 (1:30)	3	3	1	1	1	2	2	2	17.54± 1.23
7	1 (70°C)	3 (12 h)	3	3	1 (1:20)	1	1	3	3	3	2	2	2	17.72± 1.40
8	1 (70°C)	3 (12 h)	3	3	2 (1:25)	2	2	1	1	1	3	3	3	18.07± 1.41
9	1 (70°C)	3 (12 h)	3	3	3 (1:30)	3	3	2	2	2	1	1	1	18.58± 1.47
10	2 (80°C)	1 (8 h)	2	3	1 (1:20)	2	3	1	2	3	1	2	3	19.43± 1.10
11	2 (80°C)	1 (8 h)	2	3	2 (1:25)	3	1	2	3	1	2	3	1	19.71± 1.07
12	2 (80°C)	1 (8 h)	2	3	3 (1:30)	1	2	3	1	2	3	1	2	17.25± 1.31
13	2 (80°C)	2 (10 h)	3	1	1 (1:20)	2	3	2	3	1	3	1	2	19.20±1.38
14	2 (80°C)	2 (10 h)	3	1	2 (1:25)	3	1	3	1	2	1	2	3	17.93± 1.56
15	2 (80°C)	2 (10 h)	3	1	3 (1:30)	1	2	1	2	3	2	3	1	18.58± 1.36
16	2 (80°C)	3 (12 h)	1	2	1 (1:20)	2	3	3	1	2	2	3	1	17.96± 2.09
17	2 (80°C)	3 (12 h)	1	2	2 (1:25)	3	1	1	2	3	3	1	2	18.94± 2.17
18	2 (80°C)	3 (12 h)	1	2	3 (1:30)	1	2	2	3	1	1	2	3	18.24± 1.36
19	3 (90°C)	1 (8 h)	3	2	1 (1:20)	3	2	1	3	2	1	3	2	18.13± 0.78
20	3 (90°C)	1 (8 h)	3	2	2 (1:25)	1	3	2	1	3	2	1	3	18.84± 2.05
21	3 (90°C)	1 (8 h)	3	2	3 (1:30)	2	1	3	2	1	3	2	1	19.45± 0.95
22	3 (90°C)	2 (10 h)	1	3	1 (1:20)	3	2	2	1	3	3	2	1	17.18± 1.98
23	3 (90°C)	2 (10 h)	1	3	2 (1:25)	1	3	3	2	1	1	3	2	18.03± 1.00
24	3 (90°C)	2 (10 h)	1	3	3 (1:30)	2	1	1	3	2	2	1	3	16.25± 1.29
25	3 (90°C)	3 (12 h)	2	1	1 (1:20)	3	2	3	2	1	2	1	3	18.66± 1.20
26	3 (90°C)	3 (12 h)	2	1	2 (1:25)	1	3	1	3	2	3	2	1	19.04± 1.79
27	3 (90°C)	3 (12 h)	2	1	3 (1:30)	2	1	2	1	3	1	3	2	18.61± 2.02
*K*_1*j*_ b	17.40	17.99			17.88									∑488.03
*K*_2*j*_	18.58	17.81			18.48									
*K_3j_*	18.24	18.42			17.87									
*R* c	1.18	0.61			0.61									

A represented the factor of extraction temperature(°C), B represented the factor of extraction time (h), and C represented the factor of solid to liquid ratio (g: mL).

a DMSO was used as control. A negative result was defined as an inhibition zone of 10 mm. Greater than 10 mm indicated positive result of the presence of antimicrobial substance. Each value was mean and standard deviation (S.D.) of twelve replications.

b *K_ij_* = (1/9) ∑ mean inhibition zone of *L. japonica* at factor *j* (*j*  =  A, B, C).

c *R_ij_* = max {*K_ij_*}−min {*K_ij_*}, *j* and *i* mean factor and setting level here, respectively.

Because interactions between factors are complex, only low–order interactions were analyzed while high–order (three–, four–, and five–order) interactions were neglected. In Table 3, the term ‘‘interaction’’, indicated by inserting the ‘‘×’’ symbol between the two interacting factors, is used to describe the condition in which the effect of one factor’s influence upon the result is dependent on the condition of the other factor. In Table 3, if the significant level, α, was 0.05, then A (temperature), and A×B (interaction of temperature and extraction time) have significant influence on the antimicrobial activity of *L. japonica*. Therefore, factor A and the interaction A×B were regarded as a dependent factor and interaction in the extraction of antimicrobial substances. B (extraction time), C (solid to liquid ratio) and interactions A×C, B×C, were regarded as independent factors and interactions. As seen from the result in Table 2, the factors that gave the optimal extraction level were A_2_B_3_C_2_, in other words, the optimum conditions for extracting antimicrobial substances from *L. japonica* were 80°C, an extraction time 12 h, and a solid to liquid ratio of 1∶25

**Table 3:** Results of variance (ANOVA) analysis.

Source	SS	*df*	MS	*F* a	Significance*^b^*
A	6.67	2	3.34	5.14	*
A×B	8.88	4	2.22	3.42	*
Error	12.91	20	0.65		
Total	28.46	26			

a Significant parameter, *F*_0.05_ (2, 20) = 3.49, *F*_0.05_ (4, 20) = 2.87. *^b^* *indicate significant different.

### Determination of the major inhibitory substances

**Figure 3:**
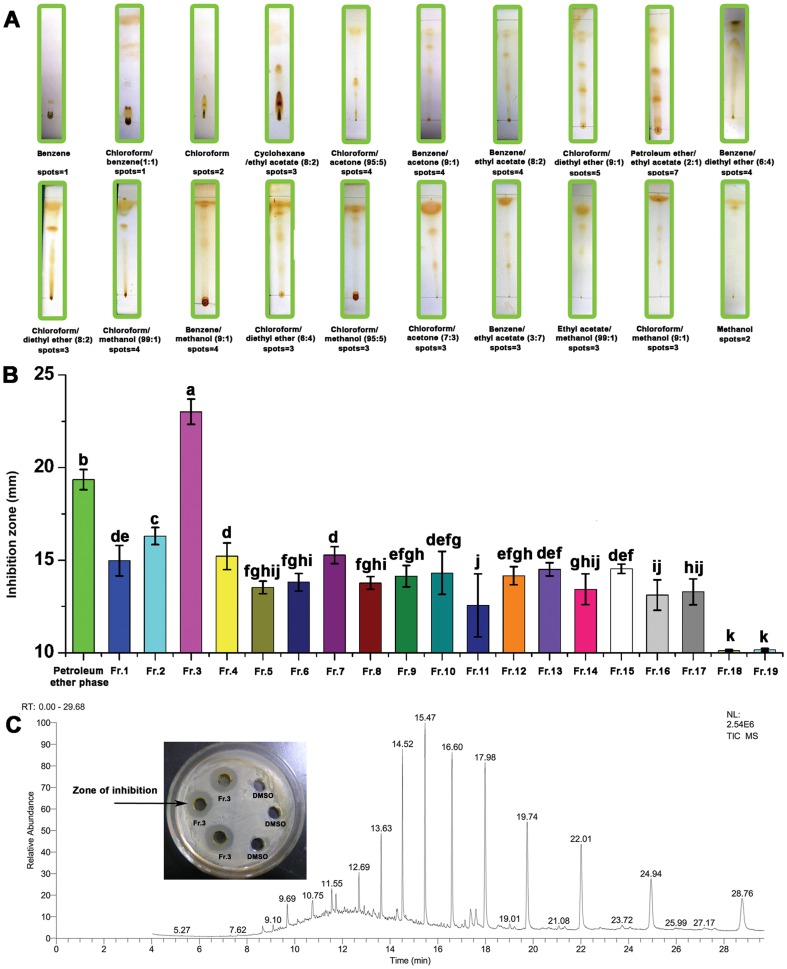
Major inhibitory substance determination. Thin layer chromatography (TLC) pattern of petroleum ether partition from *L. japonica* alcoholic extracts developed in twenty solvent systems (A). Inhibitory activities of nineteen fractionations isolated from the petroleum ether partition (B). Gas chromatography–mass spectroscopy (GC–MS) of Fractionation three (C). A negative result is defined as an inhibition zone of 10 mm, while an inhibition zone of greater than 10 mm indicates a positive result in the presence of inhibitory substance. Different letters indicate a significant difference (*P*<0.05; ANOVA and Duncan’s multiple range test). Bars represent the mean ± standard deviation. Each experiment was replicated five times. Fr., Fractionation.

At 1 mg/mL, the petroleum ether fraction showed the highest antimicrobial activity. Hence, the petroleum ether fraction was subjected to subsequent analysis. To select a mobile phase for silica gel column chromatography, a thin layer chromatography (TLC) method was performed. Among the twenty solvent systems tested, petroleum ether/ethyl acetate (2∶1) could be used to separate up to seven spots (Figure 3A). Therefore, we selected petroleum ether/ethyl acetate as the mobile phase for silica gel column chromatography. The petroleum ether fraction was subjected to silica gel column chromatography with petroleum ether/ethyl acetate as the eluent, and the polarity was slowly increased to pure ethyl acetate. Nineteen fractionations were isolated from the petroleum ether fraction, and Fractionation three (Fr.3) showed the highest (*P*<0.05) antimicrobial activity against *C. michiganense* subsp. *sepedonicum* (Figure 3B). Fr.3 obtained from the first step of column chromatography (petroleum ether/ethyl acetate 100: 3 (v/v)) was subjected to a second round of chromatography. However, the sub–fractionations obtained from the second round of chromatography did not show higher inhibitory activities against *C. michiganense* subsp. *sepedonicum*, than Fr.3. Hence, Fr.3 was subjected to subsequent chemical composition analysis.

Analysis of chemical composition by gas chromatography–mass spectroscopy (GC–MS) identified a total of 26 components, representing 94.48% of Fr.3 (Table 4, Figure 3C). These components were classified into six different classes, including 11 alkanes, 5 esters, 5 acids, 3 alcohols, 1 phenol and 1 anhydride. The major components were pentacosane (11.97%), heptacosane (10.71%), tetratriacontane (10.22%), hentriacontane (10.07%), nonacosane (9.68%), octacosane (7.57%), hexatriacontane (7.32%), tetratetracontane (5.88%) and docosane (4.04%). In summary, Fr.3 contained high amounts of alkanes (80.97%), esters (5.24%), acids (4.87%) and alcohols (2.21%).

**Table 4:** The main constituents of Fractionation three determined by gas chromatography–mass spectroscopy (GC–MS) analysis.

Peak number	Compound a	Retention time (min)	Molecular formula	Chemical composition (%) *^b^*
1	Tert-hexadecanethiol	9.10	C_16_H_34_S	0.35
2	3-Ethyl-5-(2-ethylbutyl) octadecane	9.69	C_26_H_54_	1.64
3	Dodeceny succinicanhydride	10.13	C_16_H_26_O_3_	0.32
4	1-Phenylpentadecane	10.74	C_21_H_36_	1.87
5	1-Heptatriacotanol	11.30	C_37_H_76_O	0.68
6	Diisononyl phthalate	11.56	C_26_H_42_O_4_	1.63
7	Gibberellic acid	11.73	C_19_H_22_O_6_	0.98
8	Linoleic acid ethyl ester	12.09	C_20_H_36_O_2_	0.47
9	Benzenepropanoic acid, 4-hydroxy-, [(4S)-2,2-dimethyl-1,3-dioxolan-4-yl]methyl ester	12.54	C_15_H_20_O_5_	0.63
10	Alatolide	12.69	C_19_H_26_O_6_	2.03
11	Methyl abietate	12.91	C_21_H_32_O_2_	0.48
12	Ursodeoxycholic acid	13.08	C_24_H_40_O_4_	0.42
13	Docosane	13.63	C_22_H_46_	4.04
14	Squalene-2, 3-diol	14.30	C_30_H_52_O_2_	1.18
15	Octacosane	14.52	C_28_H_58_	7.57
16	Hentriacontane	15.47	C_31_H_64_	10.07
17	Heptacosane	16.60	C_27_H_54_	10.71
18	2,2'-Methylenebis(6-tert-butyl-4-methylphenol)	17.14	C_23_H_32_O_2_	0.87
19	3,3,3-Triphenylpropionic acid	17.38	C_21_H_18_O_2_	2.21
20	Pentacosane	17.98	C_25_H_52_	11.97
21	Abietic acid	18.54	C_20_H_30_O_2_	0.85
22	(Z,Z,Z)-9,12,15-Octadecatrienoic acid	19.02	C_18_H_30_O_2_	0.41
23	Tetratriacontane	19.75	C_34_H_70_	10.22
24	Nonacosane	22.01	C_29_H_60_	9.68
25	Hexatriacontane	24.94	C_36_H_74_	7.32
26	Tetratetracontane	28.76	C_44_H_90_	5.88

a All constituents were tentatively identified. *^b^* Relative area (peak area relative to the total peak area).

### Investigation of the Inhibition Mechanism

#### Minimum inhibitory concentration (MIC) determination

**Figure 4:**
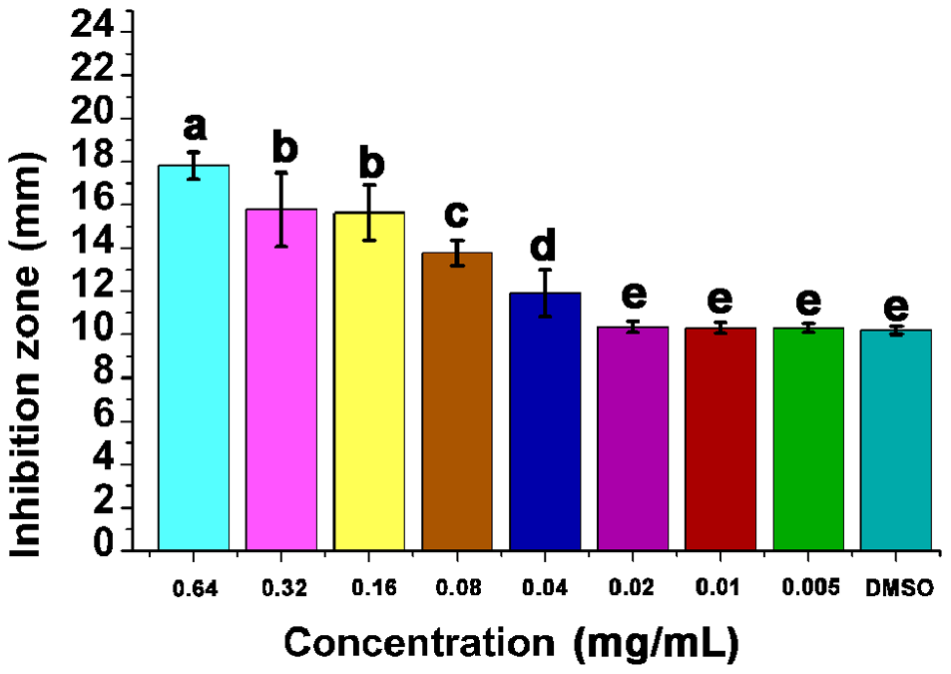
Inhibitory effects of Fractionation three at different concentrations against *C. michiganense* subsp. *sepedonicum*. DMSO was used as the control. A negative result is defined as an inhibition zone of 10(*P*<0.05; ANOVA and Duncan’s multiple range test). Bars represent the mean ± standard deviation. Each experiment was replicated four times.

The MIC of Fr.3 against *C. michiganense* subsp. *sepedonicum* was determined by the agar diffusion method (ADM). As shown in Figure 4, Fr.3 was effective in inhibiting the growth of *C. michiganense* subsp. *sepedonicum* when tested at 0.64 mg/mL, 0.32 mg/mL, 0.16 mg/mL, 0.08 mg/mL and 0.04 mg/mL, compared to the control (*P*<0.05; Figure 4). There were no statistically significant (*P*<0.05) differences between the 0.02 mg/mL, 0.01 mg/mL, 0.005 mg/mL samples and the negative control, dimethyl sulphoxide (DMSO). These data indicated that Fr.3 exhibited concentration–dependent inhibition of *C. michiganense* subsp. *sepedonicum* growth. Fr.3 at 0.04 mg/mL was enough to inhibit the growth of *C. michiganense* subsp. *sepedonicum*. Thus, the MIC of Fr.3 against *C. michiganense* subsp. *sepedonicum* was 0.04 mg/mL.

#### Growth curve

**Figure 5:**
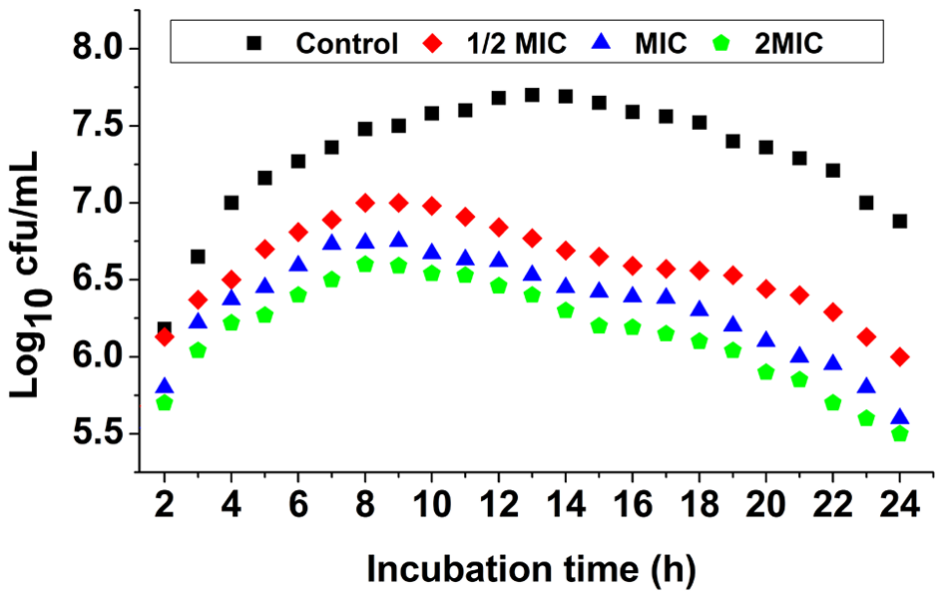
Growth curves of *C. michiganense* subsp. *sepedonicum* in the presence and absence of Fractionation three. MIC, Minimum inhibitory concentration.

The result of bactericidal kinetic assay is presented as a log_10_ cfu/mL change (Figure 5). Compared to the control, a lower concentration of Fr.3 (1/2MIC concentration) demonstrated a weaker inhibitory effect during a 24 h period. At the MIC and 2MIC, Fr.3 suppressed the growth of *C. michiganense* subsp. *sepedonicum* significantly following a 2 h exposure. A clear correlation was observed between the concentration of Fr.3 and its inhibitory activity.

#### Scanning electron microscopy (SEM) and transmission electron microscopy (TEM) observations

**Figure 6:**
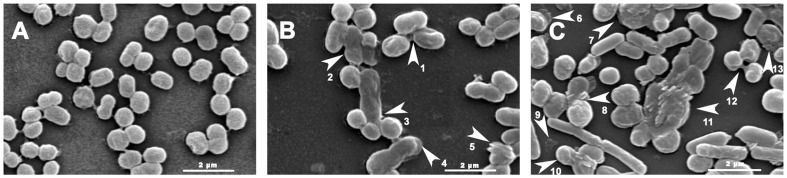
Scanning electron micrographs (SEM) of *C. michiganense* subsp. *sepedonicum* cells. Untreated (A, 10000×); treated with Fractionation three at the MIC concentration (B, 10000×), and treated at 2MIC concentration (C, 10000×). Extracellular leakage of cell content (1, 6, 12, 13), cells gathered into “clumps”(2, 7, 8, 11, 12), misshapen cells (3), blebs of damaged cells (4), roughened cell surfaces (5), collapsed surfaces (7, 8, 10, 11, 12), fragmentation (9), swollen cells (7, 8, 10, 12), disrupted cytoplasmic membrane (8, 10, 11, 12), and cell lysis (9). MIC, Minimum inhibitory concentration.

**Figure 7:**
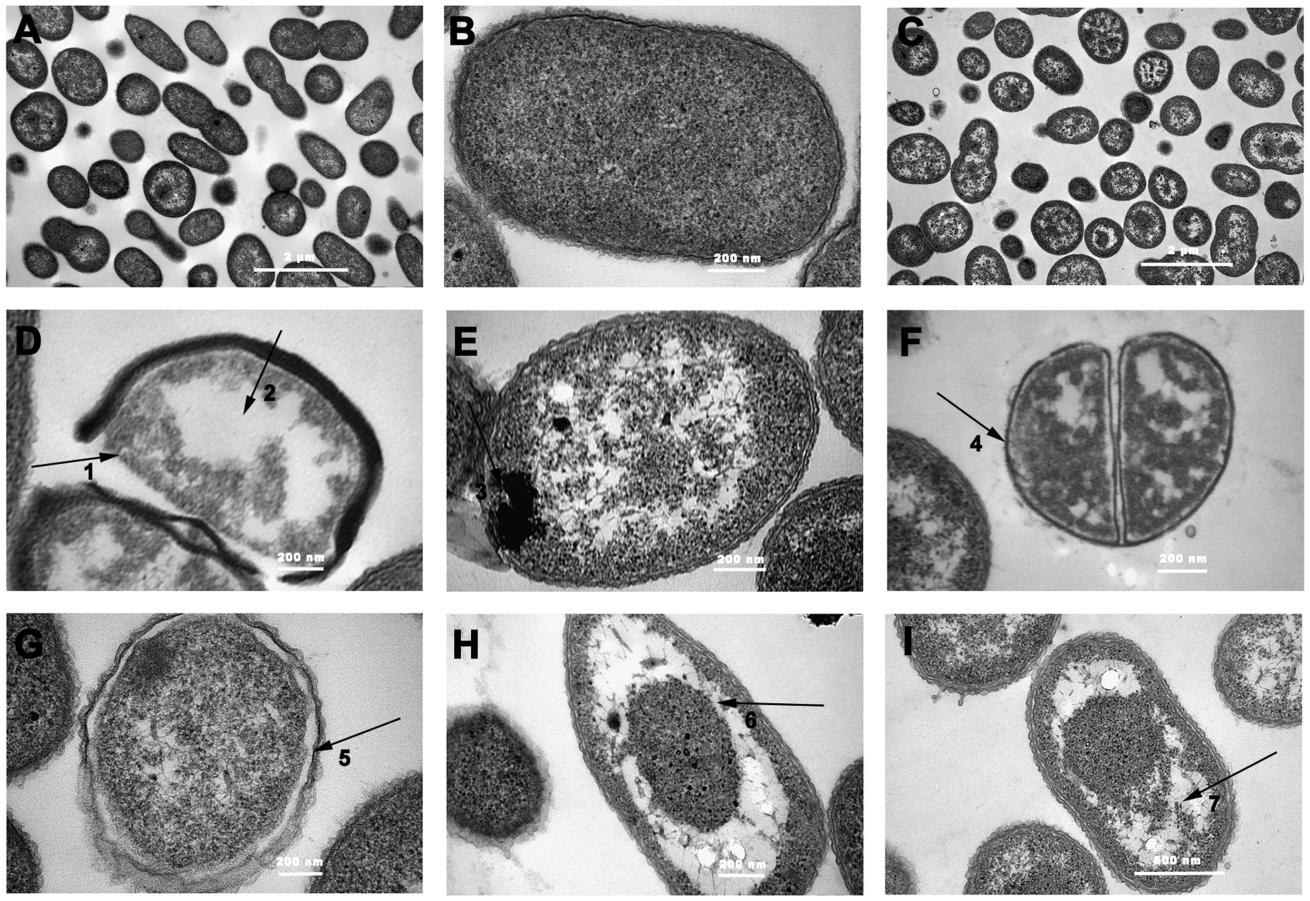
Transmission electron micrographs (TEM) of *C. michiganense* subsp. *sepedonicum* cells. Untreated (A, 20000×; B, 120000×); treated with Fractionation three at the MIC concentration (C, 20000×; D, F, G, H, 100000×; E, 120000×; I, 80000×). Disintegration of cell wall (1, 3), cytoplasm separated from cell wall and cell membrane (4, 5), large vacuoles inside the cells (2, 6, 7). MIC, Minimum inhibitory concentration.

SEM and TEM are suitable tools for investigation of the morphology and microstructure of *C. michiganense* subsp. *sepedonicum* [21]. A SEM study was used to examine any surface alterations and or general morphological changes of *C. michiganense* subsp. *sepedonicum* after exposure to Fr.3. All the control cells of *C. michiganense* subsp. *sepedonicum* were generally smooth–walled bodies, spherical in shape and were separated from each other. (Figure 6A). Microscopic observation of *C. michiganense* subsp. *sepedonicum* exposed to Fr.3 at the MIC (Figure 6B) showed severe changes compared to the control. Some damaged cells showed intracellular constituent leakage (Figure 6B: 1), while others were misshapen (Figure 6B: 3). The damaged cell surfaces became roughened (Figure 6B: 5) and were decorated with blebs (Figure 6B: 4). In addition, some cells were observed to clump (Figure 6B: 2). It was clearly observed that the number of the damaged cells treated with Fr.3 (2MIC concentration) was significantly greater than that in the control (Figure 6C). Damaged cells showed significant leakage (Figure 6C: 6, 12, 13), surface collapse (Figure 6C: 7, 8, 10, 11, 12), were fragmented (Figure 6C: 9), clumped (Figure 6C: 7, 8, 11, 12), swollen (Figure 6C: 7, 8, 10, 12), had a disrupted cytoplasmic membrane (Figure 6C: 8, 10, 11, 12), or were completely lysed (Figure 6C: 9). The internal structure of *C. michiganense* subsp. *sepedonicum* treated with Fr.3 was observed with TEM. The untreated *C. michiganense* subsp. *sepedonicum* cells retained their connatural morphology (regular outlined cell wall, plasma lemma lying closely to the cell wall, and regularly distributed cytoplasm) and seemed to be normal (Figure 7A–B). In contrast, *C. michiganense* subsp. *sepedonicum* treated with Fr.3 (MIC concentration) exhibited a wide range of abnormalities (Figure 7 C–I). At the MIC, the treated cells contained small vacuoles (Figure 7C). At certain locations, the leakage of intracellular content because of cell wall and cell membrane damage was also observed (Figure 7D: 1; Figure 7E: 3). The cytoplasm was separated from the cell wall and cell membrane (Figure 7F: 4; Figure 7G: 5). Furthermore, the damaged cells also contained some large vacuoles (Figure 7D: 2; Figure 7H: 6; Figure 7I: 7), which were rarely observed in control cells.

#### Integrity of the cell wall and cell membrane

**Figure 8:**
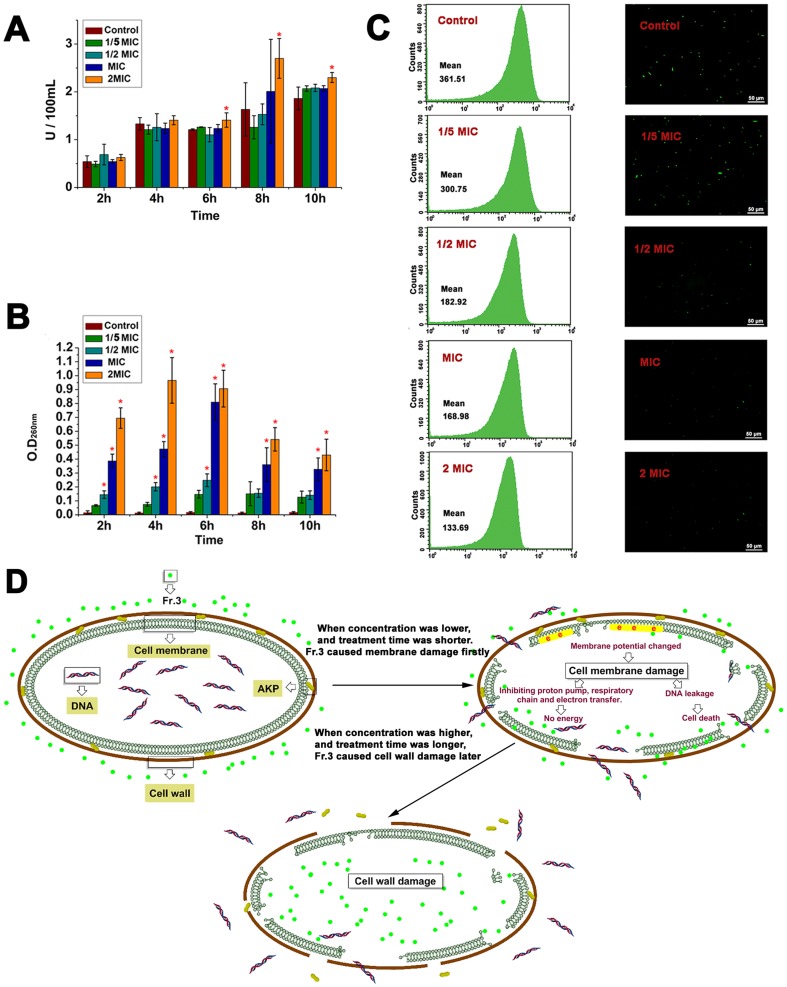
Effects of Fractionation three on the cell wall and cell membrane permeability. Effect of Fractionation three on the alkaline phosphatase (AKP) activity of *C. michiganense* subsp. *sepedonicum* (A). Effect of Fractionation three on the leakage of nucleotides from *C. michiganense* subsp. *sepedonicum* (B). Cell membrane depolarization in *C. michiganense* subsp. *sepedonicum* cells treated with Fractionation three (C). Proposed mechanism of cell wall and cell membrane disruption by Fractionation three treatment (D). Different letters indicated a significant difference (*P*<0.05; ANOVA and Duncan’s multiple range test). Bars represent the mean ± standard deviation. Each experiment was replicated three times. Analysis of cell membrane potential by flow cytometry (C left panel) and fluorescence microscopy (C right panel, 20×). MIC, Minimum inhibitory concentration; Fr.3, Fractionation three.

Alkaline phosphatase (AKP) was used to determine the effect of Fr.3 on the cell wall. As shown in Figure 8A, there were no significant differences between the control, and treatments at 1/5MIC, 1/2MIC, or the MIC. However, a significant (*P*<0.05) difference in AKP activity was observed when the cells were treated with Fr.3 at 2MIC for 6 h, 8 h and 10 h. The result indicated that Fr.3 had an effect on the cell wall permeability. Figure 8B shows the leakage of nucleotides from the *C. michiganense* subsp. *sepedonicum* cells treated with Fr.3. There were no significant differences between the control and treatment at 1/5MIC. However, when the concentration of Fr.3 increased to the MIC and 2MIC, OD values at 260 nm were all significantly (*P*<0.05) higher than the control. A significant correlation was found between the concentration of Fr.3 and OD value at 260 nm. The result indicated that Fr.3 had an effect on the membrane permeability of the *C. michiganense* subsp. *sepedonicum*, and the effect was dose–dependent. In Figure 8C, dissipation of the membrane potential by Fr.3 treatment was clearly evident from flow cytometry analyses. After 8 h, the fluorescence value of the control was 361.51, whereas it was 300.75 for the 1/5MIC group, 182.92 for the 1/2MIC group, 168.98 for the MIC group, and was only 133.69 for the 2MIC group. The result clearly indicated that the membrane potential is disrupted after Fr.3 treatment.

#### Effects of Fr.3 on reactive oxygen species (ROS), superoxide dismutase (SOD), and catalase (CAT)

**Figure 9:**
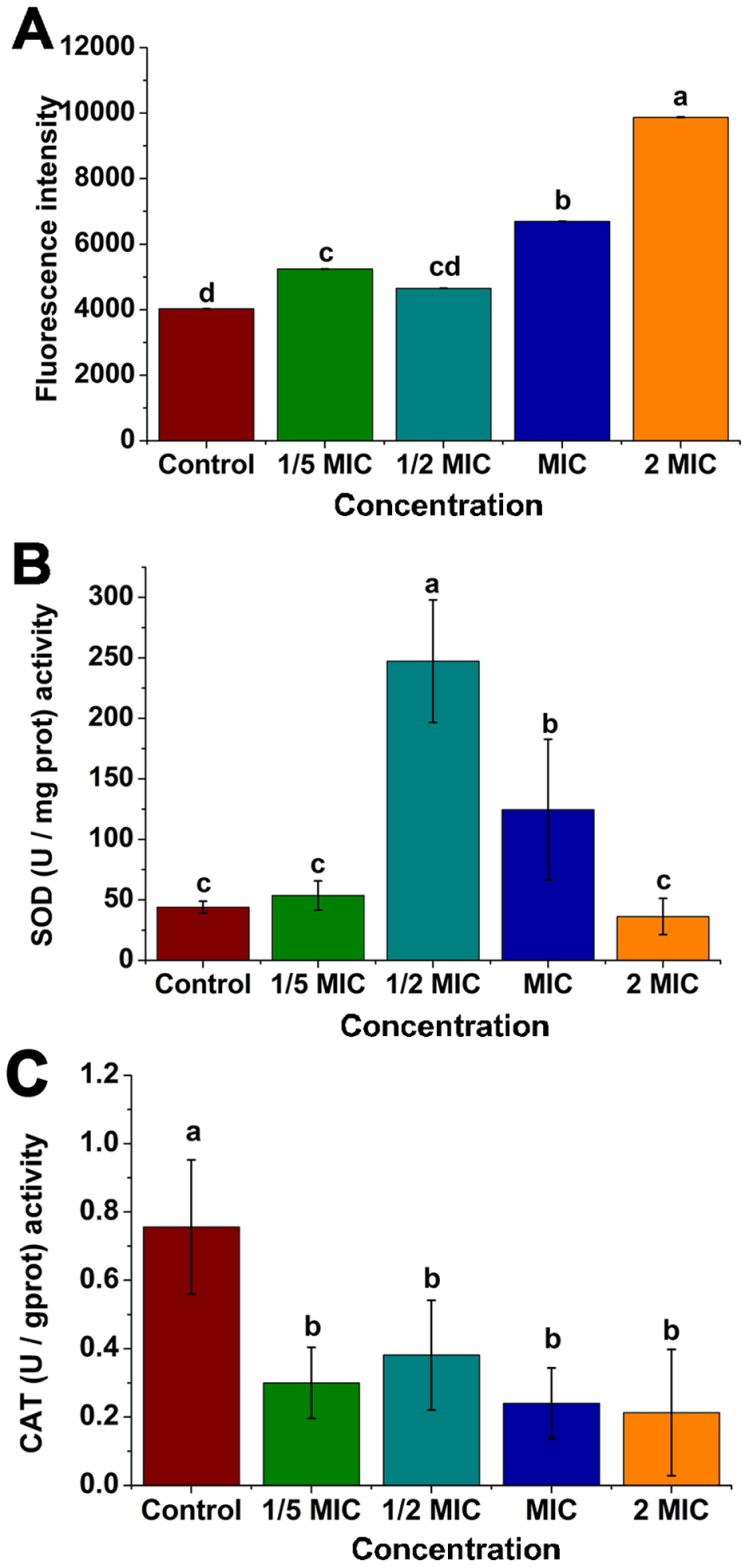
Measurement of reactive oxygen species (A), superoxide dismutase (B) and catalase (C). Cells without Fractionation three treatment were used as the control. Different letters indicated a significant difference (*P*<0.05; ANOVA and Duncan’s multiple range test). Bars represent the mean ± standard deviation. Each experiment was replicated three times. MIC, Minimum inhibitory concentration; ROS, reactive oxygen species; SOD, superoxide dismutase; CAT, catalase.

Compared with the control group, higher (*P*<0.05) levels of ROS were found in the MIC and 2MIC groups (Figure 9A). An increase in the concentration led to greater SOD activity, and the highest SOD activity with a significant (*P*<0.05) difference was observed for the 1/2MIC group. However, increasing the concentration to the 2MIC did not improve the SOD activity (Figure 9B). Treatment with Fr.3 (1/5MIC, 1/2MIC, MIC, and 2MIC) resulted in a statistically significant (*P*<0.05) decrease in the CAT activity compared to the control (Figure 9C).

#### Fr.3 increases the cytosolic Ca^2+^ level in *C. michiganense* subsp. *sepedonicum*

**Figure 10:**
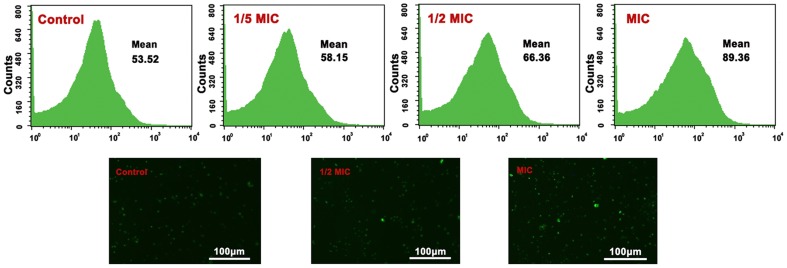
Cytosolic Ca^2+^ concentration in *C. michiganense* subsp. *sepedonicum* cells treated with Fractionation three. Analysis of Ca^2+^ concentration in *C. michiganense* subsp. *sepedonicum* cells by flow cytometry (upper panel) and fluorescence microscopy (lower panel, 10×). MIC, Minimum inhibitory concentration.

Fluo3–AM is a Ca^2+^ indicator which, when combined with Ca^2+^, yields a fluorescence output that corresponds to intracellular Ca^2+^ [22]. As shown in Figure 10, the fluorescence intensity of the control group was 53.52, whereas it was 58.15 for the 1/5MIC group, 66.36 for the 1/2MIC group, and 89.36 for the MIC group, respectively. Intracellular Ca^2+^ was increased after the treatment with Fr.3.

#### Inhibition of cell respiration by Fr.3

**Figure 11:**
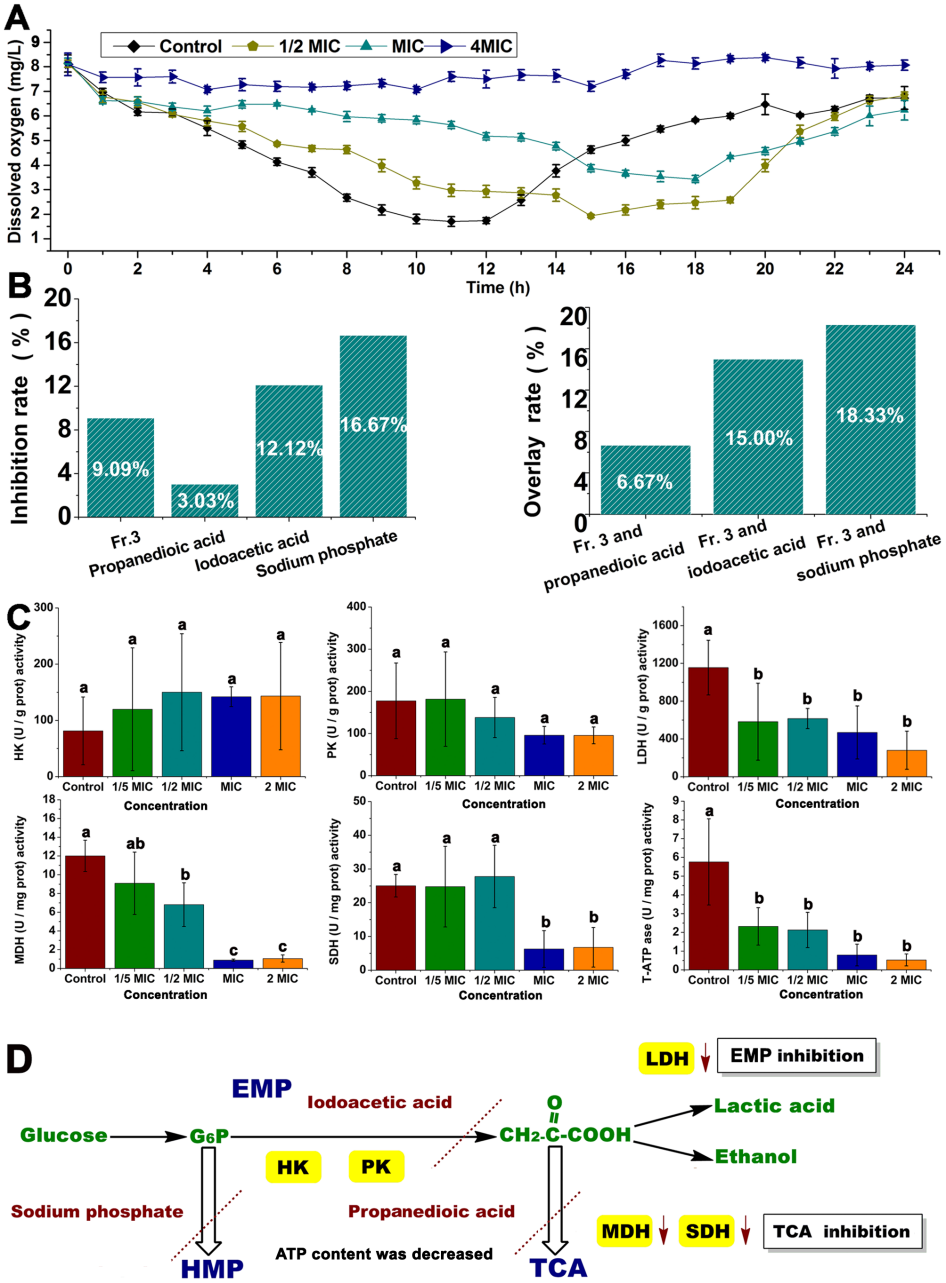
Effect of Fractionation three on the cellular respiration of *C. michiganense* subsp. *sepedonicum*. Determination of dissolved oxygen (A). Determination of inhibition rate and overlay rate (B). Effect of Fractionation three on hexokinase (HK), pyruvate kinase (PK), lactate dehydrogenase (LDH), malate dehydrogenase (MDH), succinate dehydrogenase (SDH) and total ATPase (T–ATPase) activities (C). Proposed mechanism of the inhibitory effect of Fractionation three on respiration (D). Cells without Fractionation three treatment were used as the control. Different letters indicated a significant difference (*P*<0.05; ANOVA and Duncan’s multiple range test). Bars represent the mean ± standard deviation. Each experiment was replicated three times. EMP, glycolytic pathway; TCA, tricarboxylic acid cycle; HMP, pentose phosphate pathway; HK, hexokinase; PK, pyruvate kinase; LDH, lactate dehydrogenase; MDH, malate dehydrogenase; SDH, succinate dehydrogenase; G-6-P, glucose-6-phosphate; MIC, Minimum inhibitory concentration; Fr.3, Fractionation three.

As shown in Figure 11A, the dissolved oxygen of the control group significantly decreased within the initial 8 h. When the control bacteria entered into stationary phase, the dissolved oxygen decreased more slowly. The dissolved oxygen increased again after the control bacteria entered into the decline phase. However, at a lower concentration of Fr.3 (1/2MIC and MIC), the curve of dissolved oxygen changed gradually. When Fr.3 was 4MIC, there was almost no change in dissolved oxygen over 24 h.

The extent of inhibition of respiration provided by Fr.3 was compared with that from 3 typical inhibitors. The inhibition rates were ranked as follows: sodium phosphate > iodoacetic acid > Fr.3 > propanedioic acid. The overlay rates of the standard inhibitors and Fr.3 were ranked as follows: overlay rate of Fr.3 and sodium phosphate > overlay rate of Fr.3 and iodoacetic acid > overlay rate of Fr.3 and propanedioic acid (Figure 11B).

The effect of Fr.3 on hexokinase (HK), pyruvate kinase (PK), lactate dehydrogenase (LDH), malate dehydrogenase (MDH), succinate dehydrogenase (SDH) and total ATPase (T–ATPase) activities in *C. michiganense* subsp. *sepedonicum* are presented in Figure 11C. HK and PK activities showed no significant differences between the control and the 1/5MIC, 1/2MIC, MIC and 2MIC groups. Activities of LDH and ATP decreased sharply (*P*<0.05) compared with the control. The MDH activity in *C. michiganense* subsp. *sepedonicum* significantly (*P*<0.05) decreased, when the concentration of Fr.3 increased to 1/2MIC, MIC and 2MIC. When Fr.3 was treated at MIC and 2MIC, the SDH activity was significantly (*P*<0.05) decreased in *C. michiganense* subsp. *sepedonicum*.

#### Bacterial proteins

**Figure 12:**
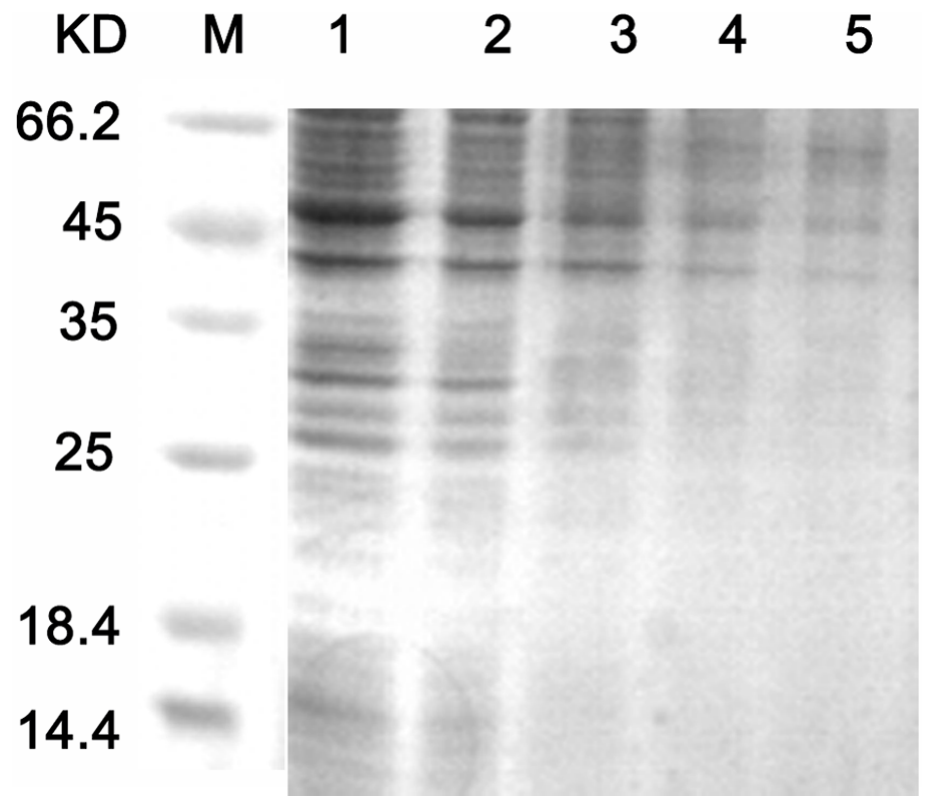
Sodium dodecyl sulfate polyacrylamide gel electrophoresis (SDS–PAGE) analysis of *C. michiganense* subsp. *sepedonicum*. Lane 1, untreated *C. michiganense* subsp. *sepedonicum*. Lane 2, *C. michiganense* subsp. *sepedonicum* cells treated with Fractionation three at 1/2MIC concentration for 8 h. Lane 3, *C. michiganense* subsp. *sepedonicum* cells treated with Fractionation three at the MIC concentration for 8 h. Lane 4, *C. michiganense* subsp. *sepedonicum* cells treated with Fractionation three at 2MIC concentration for 8 h. Lane 5, *C. michiganense* subsp. *sepedonicum* cells treated with Fractionation three at 4MIC concentration for 8 h. Lane M, marker.

Protein electrophoresis bands from normal *C. michiganense* subsp. *sepedonicum* cells were easily visualized. After treatment with Fr.3 (1/2MIC, MIC and 2MIC), the protein bands appeared shallow or even disappeared (14.4–35 kDa). As the concentration of Fr.3 increased to 4MIC, the disappearance of the protein bands became more distinct (Figure 12). Therefore, the inhibitory action of Fr.3 was dose–dependent.

#### Inhibition of nucleic acids

**Figure 13:**
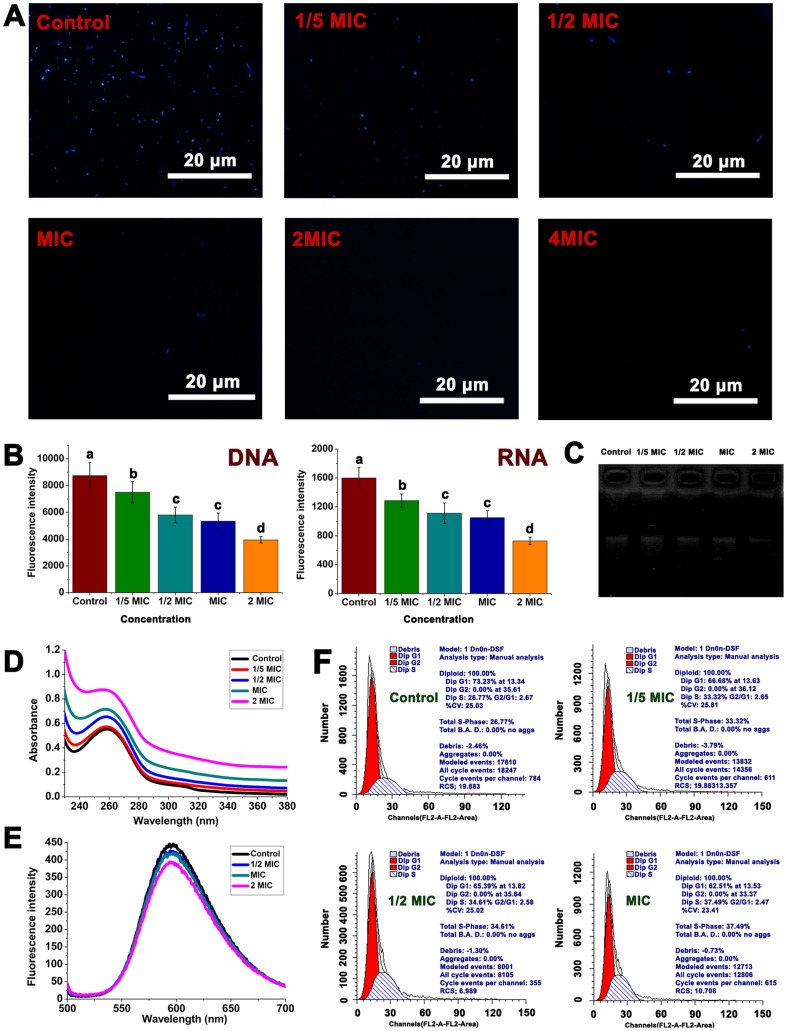
Inhibition of *C. michiganense* subsp. *Sepedonicum* nucleic acid synthesis by treatment with Fractionation three. Fluorescence micrographs of *C. michiganense* subsp. *sepedonicum* treated with Fractionation three at 1/5MIC, 1/2MIC, MIC, 2MIC, and 4MIC concentrations (A, 100×). Effect of Fractionation three on the synthesis of nucleic acid in *C. michiganense* subsp. *sepedonicum* (B). Gel retardation assays (C). UV–visible spectra of genomic DNA treated with increasing concentrations of Fractionation three (D; 1/5MIC; 1/2MIC, MIC, and 2MIC). Competitive binding of Fractionation three (1/2MIC, MIC, and 2MIC) and EB with *C. michiganense* subsp. *sepedonicum* genomic DNA (E). Flow cytometric analysis of the cell cycle (F). Cells without Fractionation three treatment were used as the control. Different letters indicated a significant difference (*P*<0.05; ANOVA and Duncan’s multiple range test). Bars represent the mean ± standard deviation. Each experiment was replicated three times. MIC, Minimum inhibitory concentration.

4,6–diamidino–2–phenylindole (DAPI) is a fluorescent dye that can penetrate the cell membrane and combine with DNA and RNA. The amount of nucleic acids in *C. michiganense* subsp. *sepedonicum* after treatment with DAPI is reflected by the fluorescence intensity. In Figure 13 (A), a fluorescence micrograph of the control nucleic acids is strongly fluorescent. The fluorescence became weaker with treatment of Fr.3 at 1/5MIC, 1/2MIC, and the MIC. There was almost no fluorescence observed after treatment with 2MIC and 4MIC of Fr.3. The results presented in Figure 13 (B) showed that the quantity of DNA and RNA were significantly (*P*<0.05) reduced when the concentration ranged from 1/5MIC, 1/2MIC, MIC, to 2MIC.

To determine the molecular mechanisms of action, the DNA–binding capability of Fr.3 was evaluated using an electrophoretic gel mobility shift assay (Figure 13C). Results showed the DNA band in the control group was easily visualized. After treatment with Fr.3 (1/5MIC, 1/2MIC and MIC), the DNA bands appeared shallow and in some cases, they disappeared (2MIC). These results indicated that Fr.3 could interact with *C. michiganense* subsp. *sepedonicum* genomic DNA, and the inhibitory action was dose–dependent.

Ultraviolet (UV)–visible scans and competitive studies with ethidium bromide (EB) were conducted in order to determine the extent of DNA conformational changes upon binding Fr.3. The absorption spectra of DNA treated with Fr.3 was shown in Figure 13D. In the UV region, samples exhibited an intense absorption band between 220 nm and 380 nm that increased with increasing concentrations of Fr.3. Competitive binding studies were performed in the presence of EB in order to investigate the existence of a potential intercalation of the contents of Fr.3 to DNA. EB is one of the most sensitive fluorescent probes for DNA structure and binds to DNA via intercalation of its planar phenanthridinium ring between adjacent base pairs on the DNA double helix. Fluorescence emission is enhanced because of intercalation of EB to DNA [23]. Fluorescence spectroscopic results are presented in Figure 13E. The fluorescence intensities of EB bound to DNA at 493 nm decreased with increases in the concentration of Fr.3.

Flow cytometry showed that the *C. michiganense* subsp. *sepedonicum* cell cycle changed upon incubation with Fr.3. In the bacterial cell cycle, the I phase (DNA preparatory period) is the same as the G_1_ phase in the eukaryotic cell cycle. The R phase (DNA copy period) is the same as the S phase in the eukaryotic cell cycle. There is no G_2_ phase in the bacterial cell cycle [24]. As shown in Figure 13F, the G_2_ phase was zero. After treatment with Fr.3 at 1/5MIC for 8 h, the number of bacteria in the I phase decreased from 73.23% to 66.68% (red area in Figure 13F), while the number of cells in the R phase increased from 26.77% to 33.32% (line area in Figure 13F). When treated at 1/2MIC and the MIC, the number of bacteria in the I phase decreased to 65.39%, and 62.51% (red area in Figure 13F), while the number in the R phase increased to 34.61% and 37.49% (line area in Figure 13F).

## Discussion

In this study, *L. japonica* was shown to have antimicrobial activity against *C. michiganense* subsp. *sepedonicum*, the causal agent of bacterial ring rot of potato. Solvents are commonly used to extract antimicrobial substances from plants. In this study, we used ethanol because the ethanol extracts had the highest (*P*<0.05) antimicrobial activity against *C. michiganense* subsp. *sepedonicum*. The three factors that affected the antimicrobial substance extraction were extraction temperature (A), extraction time (B), and solid to liquid ratio (C) [25]. The effect of temperature on antimicrobial activity was studied by varying the temperature from 40 to 98°C. The data (Figure 2A) indicated that increasing the temperature enhanced diffusivity and hence, the antimicrobial activity increased with increasing temperature [26]. When the temperature was too high, ethanol volatilization was accelerated and the solid to liquid ratio was lowed, and thus the yield of antimicrobial activity was decreased. Extraction time is one of the most influential parameters in antimicrobial substance extraction. Shorter extraction times resulted in incomplete extraction while longer extraction times waste time and energy, and leads to the possibility that the antimicrobial components might decompose (Figure 2B) [27]. Seven solid to liquid ratios were investigated (Figure 2C). Because of insufficient contact between the solvent and the antimicrobial substance, it was not possible to extract the maximal amount of antimicrobial substances when the solid to liquid ratio was too low. When the solid to liquid ratio was too high, the time to concentrate would be long and the antimicrobial components might decompose [28]. An orthogonal array provides the shortest possible matrix of combinations in which all the parameters are varied to simultaneously consider their direct effect as well as their interactions. It can effectively screen out key variables by several representative experiments [29]–[33]. The results (Table 2, Table 3) revealed that factor A, and the interaction A×B had a significant (*P*<0.05) effect on the antimicrobial activity of *L. japonica*. The optimum extraction conditions for *L. japonica* were defined as below: temperature 80°C, extraction time 12 h, and a solid to liquid ratio of 1∶25.

Active compound isolation and analysis are two important steps in plant extraction. Solvent partition, TLC and column chromatography are methods used to identify inhibitory compounds. All nineteen fractionations had antimicrobial activities against *C. michiganense* subsp. *sepedonicum*. Fr.3 had the highest (*P*<0.05) inhibitory activity, and sub–fractionations obtained from Fr.3 did not have inhibitory activities higher than Fr.3. It can be concluded that the antimicrobial activity of Fr.3 against *C. michiganense* subsp. *sepedonicum* should be the result of synergy between a mixture of compounds, rather than a single compound. The components in Fr.3 might be linked together with chemical bonds. As the extract became more pure, the chemical bonds between the molecules were destroyed and the inhibitory activity decreased. GC-MS analysis revealed an abundance of alkanes, esters, acids and alcohols in Fr.3. According to the reports, we know that some algae have antimicrobial activities, and the main antimicrobial components in algae are alkanes. Zhen [34] indicated that the petroleum ether fraction of *Pachydictyon coriaceum* extracts had antimicrobial activity against *Rhizopus chinensi*, *Penicillium chrysogenum* and *Pyricularia oryzae*. Chemical analysis by GC-MS indicated that alkanes of n-C_15_∼n-C_29_ could be found in the petroleum ether fraction. Karabay-Yavasoglu [35] indicated that volatile oil of *Jania rubens* (red alga) showed antimicrobial activity against five gram-positive and four gram-negative bacteria. GC-MS analysis identified 40 compounds, and docosane and tetratriacontane were the major components. It is evident from these data reported here that antimicrobial activity in algae is closely related with alkanes. Fr.3 contained high amounts of alkanes (80.97%), which might induce antimicrobial activity. In addition, Fr.3 exhibited inhibitory activities which might be attributed to the presence of abietic acid, linoleic acid ethyl ester, hexatriacontane, tetratetracontane, tetratriacontane, nonacosane, pentacosane, docosane, octacosane, hentriacontane and heptacosane. These compounds are known in plant extracts which have inhibitory activities [36]–[52].

In the present study, Fr.3 showed antimicrobial activity against *C. michiganense* subsp. *sepedonicum* even at lower concentration (0.04 mg/mL). As shown in Figure 5, the inhibitory effect of Fr.3 mainly occurred during the logarithmic phase indicating that Fr.3 inhibited the cell division of *C. michiganense* subsp. *sepedonicum*. After a 2 h exposure, the number of *C. michiganense* subsp. *sepedonicum* decreased significantly.

SEM and TEM were used to investigate possible changes in cell morphology. After treatment with Fr.3, *C. michiganense* subsp. *sepedonicum* underwent cell wall disintegration, cell membrane disruption, cell swelling, fragmentation, clumping, bleb formation, separation between the cell wall and cell membrane, formation of vacuoles, decrease in cytoplasmic materials, and cell lysis (Figure 6 and Figure 7). The ultrastructural analysis highlighted the multiple sites of action of Fr.3 in *C. michiganense* subsp. *sepedonicum*. It is believed that the active components in Fr.3 disrupted the cell wall and cell membrane of *C. michiganense* subsp. *sepedonicum* (Figure 6: 5, 7, 8, 10, 11, 12; Figure 7: 1, 3), thereby causing leakage of the bacterial cell content (Figure 6: 1, 6, 12, 13) resulting in the appearance of vacuoles (Figure 7: 2, 6, 7). Finally these changes resulted in fragmentation, misshapen cells, cell lysis and cell death (Figure 6: 3, 9, 10, 11). The distortion of the cell physical structure caused expansion (Figure 6: 7, 8, 10, 12) and destabilization of the membrane and would increase membrane fluidity, which in turn would increase passive permeability [53]. Polysaccharides that leak out of the cell would generate cell adhesions producing a ‘clump’ like shape (Figure 6: 2, 7, 8, 11, 12). Separation between the cell wall and cell membrane (Figure 7: 4, 5) was found in some damaged cells. This phenomenon might be caused by osmotic pressure changes induced by the active components in Fr.3. Thus, the use of SEM and TEM provided evidence of the antimicrobial activity of the components of Fr.3.

The cell membrane permeability analysis (Figure 8B) suggested that the action of Fr.3 (MIC and 2MIC) on the cell membrane lead to cell damage and content leakage from 2 h to 10 h after treatment. As shown in Figure 8C, the fluorescence decreased from 361.51 (control) to 133.69 (2MIC treatment). This suggested that Fr.3 caused disruption of the cell membrane by inducing depolarization. GC–MS analysis revealed that Fr.3 contained abundant lipophilic compounds (alkanes and esters). Lipophilic compounds have the ability to interact with hydrophobic structures like bacterial membranes [54]. We speculated that the active compounds of Fr.3 disrupted the cytoplasmic membrane of *C. michiganense* subsp. *sepedonicum*, thereby causing leakage of the bacterial cell content. The dysfunction and disruption of the membrane, interference with the energy generation system in the cell, and enzyme inhibition preventing substrate utilization for energy production may also lead to the death of bacterial cells [55]–[58]. AKP is an enzyme located between the cell membrane and cell wall [58]. It functions to effectively maintain the cellular osmotic pressure and cell shape. When the cell wall is intact, AKP cannot pass through the cell walls, and it is not detected in the periplasmic space. However, damage to the external cell wall layers can cause the release of AKP from the cell [59]. In the present study, significantly (*P*<0.05) higher AKP activity was only observed when concentration of Fr.3 was 2MIC and the treatment time was 6 h, 8 h and 10 h (Figure 8A). This result indicated that the cell wall of *C. michiganense* subsp. *sepedonicum* was destroyed only when the concentration was higher (2MIC) and the treatment time was longer (6 h). After a comprehensive consideration of all the results (Figure 8A, B, and C), we developed a potential mechanism to account for the antimicrobial activity of Fr.3 (Figure 8D). We speculated that the active compounds in Fr. 3 penetrated the cell walls and disrupted the cell membrane structures firstly. The cell wall was not destroyed when the concentration was lower and the treatment time was shorter. However, when the treatment time and concentration reached a certain level (2MIC and 6 h), the cell wall was then damaged.

Treatment of *C. michiganense* subsp. *sepedonicum* with Fr.3 might enhance production of reactive oxygen species (ROS; Figure 9A). Recently Kohanski et al. [60] demonstrated that the production of ROS contributes to the antimicrobial activity of bactericidal antibiotics. An earlier report suggested that the excessive accumulation of ROS within the cells can cause damage to DNA, proteins and lipids which leads to disorganization, dysfunction and damage of membranes and proteins. Thus, the ROS production by Fr.3 might trigger a cascade of events like protein carbonylation, lipid peroxidation, mitochondrial membrane depolarization and DNA fragmentation in *C. michiganense* subsp. *sepedonicum* [61], [62]. SOD and CAT play central roles in the enzymatic defense system against oxidative exposure to eliminate ROS and to reduce damaging effects [63]–[66]. The changes of SOD activity (Figure 9B) indicated that Fr.3 caused SOD to increase at lower concentrations. As the concentration of Fr.3 increased to its MIC, the ROS exceeded the ability of SOD to eliminate them, and the SOD activity was decreased. The decrease in CAT activity (Figure 9C) indicated Fr.3 might directly inhibit CAT indicating that CAT was not able to eliminate the ROS. These findings suggested that the inhibitory effect of Fr.3 was associated with oxidative damage.

Ca^2+^ plays an import role in the cell. Ca^2+^ movement is strictly regulated in healthy cells. In general, the concentration of intracellular Ca^2+^ is significantly lower than the extracellular concentration. However, when the cell membrane is seriously damaged, the inflow of extracellular Ca^2+^ can initiate an increase in intracellular Ca^2+^ concentration. The Ca^2+^ in *C. michiganense* subsp. *sepedonicum* was increased when the samples were treated with different concentrations of Fr.3 (Figure 10). This result indicated that the damaged membrane of *C. michiganense* subsp. *sepedonicum* caused the inflow of Ca^2+^. In addition, ROS induced by Fr.3 might act as second messengers to activate Ca^2+^ channels, further contributing to increases in Ca^2+^ concentration [67], [68]. On the other hand, active Ca^2+^ channels would further increase the permeability of the cell membrane.

In Figure 11A, the control cells showed a typical respiration curve. However, at lower Fr.3 concentrations, the curve of dissolved oxygen changed more gradually. When the concentration of Fr.3 was 4MIC, there was almost no change in dissolved oxygen. The result indicated that the respiration of *C. michiganense* subsp. *sepedonicum* was inhibited by Fr.3, and the effect was dose dependent. Iodoacetic acid, propanedioic acid, and sodium phosphate are three inhibitors known to inhibit the glycolytic pathway (EMP), tricarboxylic acid cycle (TCA) and pentose phosphate pathway (HMP), respectively. Calculation of the overlay rate of the known inhibitor and Fr.3 could determine which pathway was inhibited by Fr.3. When Fr.3 and the known inhibitor inhibited different respiration pathways, there should be synergy between Fr.3 and the known inhibitor, resulting in a higher overlay rate. When Fr.3 and the known inhibitor inhibited the same respiration pathway, the overlay rate of Fr.3 and the known inhibitor should be lower [69], [70]. As shown in Figure 11B, the overlay rates of the known inhibitors and Fr.3 were ranked as: overlay rate of Fr.3 and sodium phosphate > overlay rate of Fr.3 and iodoacetic acid > overlay rate of Fr.3 and propanedioic acid. This result revealed that the EMP, TCA and HMP in *C. michiganense* subsp. *sepedonicum* were all inhibited by Fr.3, and that the inhibition effect of Fr.3 on TCA was the most significant. The EMP and TCA cycle oxidize hexose to ATP and NADH, the major energy currencies of the cell [71]. Analysis of the enzyme activities of HK, PK, and LDH indicated that the EMP in *C. michiganense* subsp. *sepedonicum* was inhibited by Fr.3 (Figure 11C). HK in cells plays a key role in regulating EMP and catalyzes glucose to glucose 6–phosphate. PK, a final–stage enzyme in EMP, catalyzes the transfer of a phosphoryl group from phosphoenolpyruvate (PEP) to adenosine diphosphate (ADP), generating the substrates ATP and pyruvate for anaerobic and aerobic metabolism [72], [73]. However, even at high concentrations, Fr.3 did not change the HK and PK activities in *C. michiganense* subsp. *sepedonicum*. LDH is a key enzyme that catalyzes the conversion of pyruvic acid to lactic acid, and enables the EMP to produce enough ATP. As shown in Figure 11C, the activity of LDH decreased significantly (*P*<0.05), when the concentrations of Fr.3 were 1/5MIC, 1/2MIC, MIC and 2MIC. This result indicated that Fr.3 inhibited LDH resulting in impairment of the EMP. We analyzed the activities of MDH and SDH, two enzymes involved in the TCA cycle. MDH, a coenzyme of NADP^+^, forms NADPH by accepting hydrogen from metabolites during biosynthesis. SDH plays an important role in the cellular energy metabolism of microbes, and its activity reflects the energy metabolic status of the bacterial cell. However the SDH and MDH activities in *C. michiganense* subsp. *sepedonicum* were all inhibited by Fr.3 at higher concentrations (Figure 11C). Thus, the production of essential amino acids in *C. michiganense* subsp. *sepedonicum* were decreased because the TCA cycle which is necessary to provide amino acids as carbon sources, was inhibited by Fr.3 [74]. After treatment with Fr.3, ATP was also decreased. One reason may be that the NADH associated with the TCA cycle was reduced or that the ATPase activity was inhibited by Fr.3 because of the increased permeability of the cytoplasmic membrane (Figure 11C). Since the respiratory chain of bacteria is located in the cell membrane, contact with the antibacterial agent destroyed the membrane structure and disrupted the function of the enzyme system in the respiratory chain [75]. Taken together, we present a theory that explains the inhibitory effect of Fr.3 on respiration in Figure 11D.

The result of the sodium dodecyl sulfate polyacrylamide gel electrophoresis (SDS–PAGE) assay showed that the total proteins in *C. michiganense* subsp. *sepedonicum* decreased following treatment with Fr.3. In addition, some protein bands even disappeared (Figure 12). We speculated that Fr.3 could inhibit protein synthesis or control gene expression or that a substantial amount of protein leaked out of the bacteria following membrane disruption. The mechanism of protein breakdown remains unclear and is a topic for future study.

Figure 13 showed that Fr.3 effectively inhibited the synthesis of nucleic acid in *C. michiganense* subsp. *sepedonicum*, resulting in a decrease in DNA and RNA (Figure 13A, B). Gel retardation analysis (Figure 13C) showed that Fr.3 could bind to DNA. This result suggested that Fr.3 could directly interact with *C. michiganense* subsp. *sepedonicum* genomic DNA. One possible mechanism of antimicrobial action of Fr.3 was related to its inhibition of metabolic pathways by blocking or reducing DNA replication and/or transcription through binding DNA [76]. In order to clarify the molecular mechanism of the DNA damage and the intracellular target of Fr.3, UV–visible absorption changes and a competitive assay employing EB were examined. The changes observed in the UV spectra may give evidence of the existing interaction mode. Generally, hyperchromism indicates that the complex binds to the negatively charged phosphate backbone at the periphery of the DNA, causing damage to the DNA double helix [77], [78]. On the other hand, hypochromism and red shift indicate a conformational change of the DNA double helix. The changes observed in the UV spectra of the DNA after mixing it with Fr.3 (increase of absorption) indicated that Fr.3 might interact with DNA by the direct formation of a new complex with double helical DNA, causing double helix structural damage. The DNA double helix possesses many hydrogen bonding sites which are accessible both in the minor and major grooves, and it is possible that the components of Fr.3 might bond with DNA through hydrogen bonds, which in turn, may contribute to the hyperchromism observed in the absorption spectra [79]. Competitive binding study with EB has been employed to study the interactions involved in DNA complex formation in order to investigate a potential intercalative binding mode. EB does not show any appreciable emission in buffer solution due to fluorescence quenching of the free EB by the solvent molecules [80]. On addition of DNA, its fluorescence intensity is highly enhanced because of its strong intercalation between the adjacent DNA base pairs [81]. Addition of a second molecule, which binds to DNA more strongly than EB, can decrease the DNA–induced EB emission [80]. The intensity of the emission band at 493 nm of the DNA–EB system significantly decreased (Figure 13E) in *C. michiganense* subsp. *sepedonicum* genomic DNA, which indicated the competition of Fr.3 components with EB in binding to DNA. The quenching of DNA–EB fluorescence suggested that Fr.3 components prevented EB from inserting into the DNA and Fr.3 could interact with DNA by intercalation [81]. The cell cycle can be thought of as a circuit of regulatory components which, by enabling an efficient flow of information, triggers events critical for cellular reproduction [82]. The results (Figure 13F) indicated that the population of the treated cells at the I phase dropped to 66.68% (1/5MIC), 65.39% (1/2MIC) and 62.51% (MIC) respectively, compared with the control (73.23%). We speculated that the components of Fr.3 inhibited RNA or protein which is related to cell division during the I phase. From Figure 13F, we know that the cell population at R phase increased to 33.32% (1/5MIC), 35.64% (1/2MIC) and 37.49% (MIC), respectively, compared with the control (26.77%). This indicated that Fr.3 disrupted R phase rather than I phase, causing most cells to remain in R phase. However, the results of the bactericidal kinetic assay revealed that inhibitory effect of Fr.3 occurred mainly during the logarithmic phase where the number of *C. michiganense* subsp. *sepedonicum* decreased significantly. These results indicated that Fr.3 led the cell population to arrest at R phase, with few cells passing through R phase into the cell division phase, finally resulting in a decrease in the number of *C. michiganense* subsp. *sepedonicum*. According to these results, we speculated that Fr.3 components disrupted the normal cell cycle of the bacteria, sequentially inhibiting the growth of the bacteria, and leading to cell lysis.

**Figure 14:**
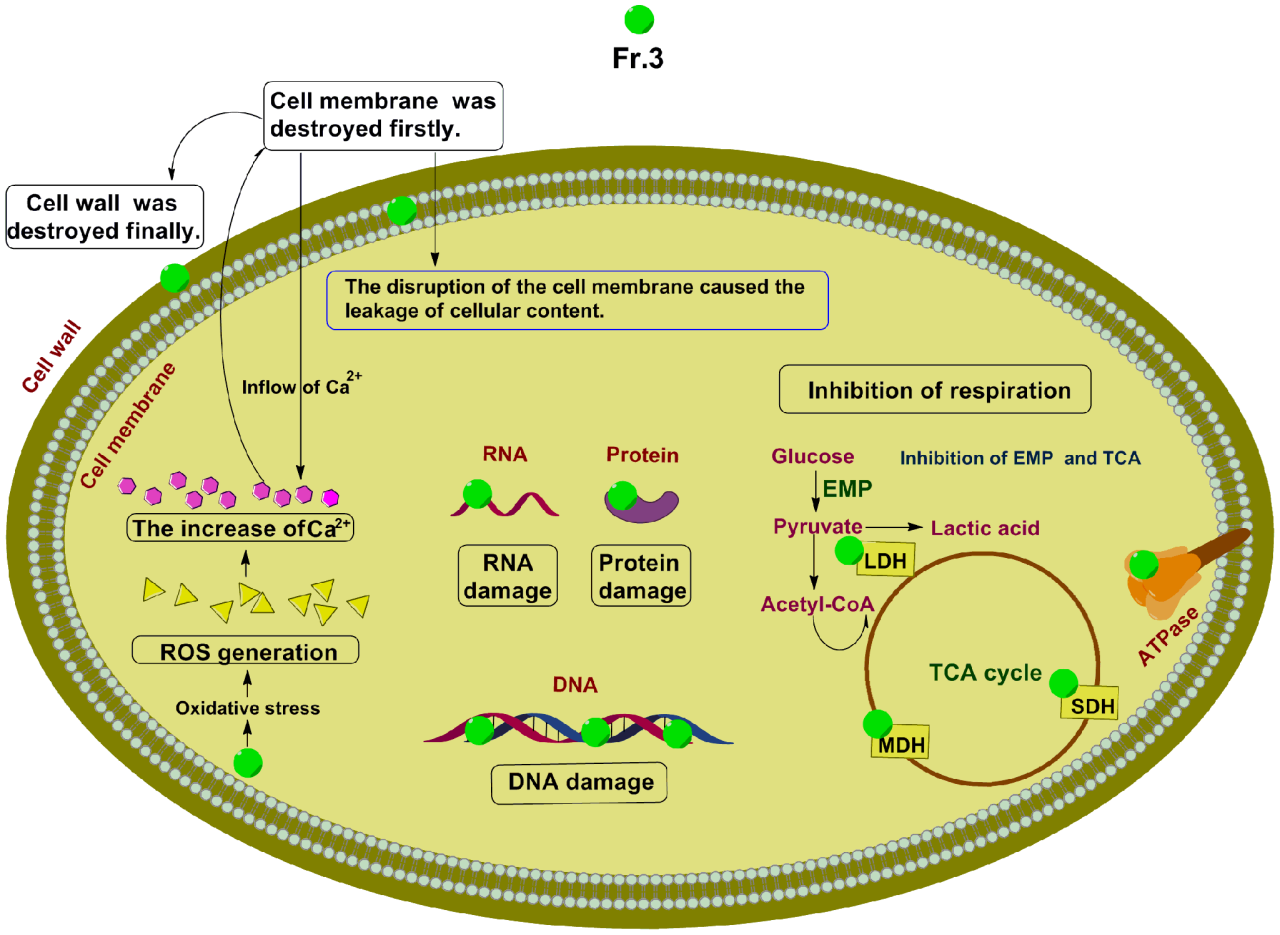
Proposed mechanism of antimicrobial activity of Fractionation three against *C. michiganense* subsp. *sepedonicum*. Arrowheads represent activating events. LDH, lactate dehydrogenase; MDH, malate dehydrogenase; SDH, succinate dehydrogenase; EMP, glycolytic pathway; TCA, tricarboxylic acid cycle; ROS, reactive oxygen species; Fr.3, Fractionation three.

Based on the present research, a schematic model of the proposed mechanism of Fr.3 is described in Figure 14. The active substances in Fr.3 resulted in loss of outer membrane integrity, causing outer membrane damage. The disruption of the cell membrane caused the leakage of cellular content, inhibition of the proton pump, respiratory chain, electron transfer and oxidative phosphorylation. A massive loss of intracellular ATP might induce a series of structural, biochemical, and functional alterations sufficient to trigger irreversible necrotic cellular pathways [83]. Secondly, Fr.3 treatment enhanced the production of ROS. The damaged cell membrane and ROS production increased Ca^2+^ in *C. michiganense* subsp. *Sepedonicum*. The activated Ca^2+^ channels would further increase the permeability of the cell membrane. In addition, Fr.3 also inhibited the EMP pathway and TCA cycle and the respiration was also inhibited by Fr.3. Thirdly, active components inhibited protein and nucleic acid synthesis, disrupting the normal cycle of DNA replication. Fr.3 could directly bind with *C. michiganense* subsp. *sepedonicum* genomic DNA, causing double helix structural damage. All these factors could result in cell decomposition and eventually death. These findings indicate that *L. japonica* extract has a significant potential for inhibiting *C. michiganense* subsp. *sepedonicum*.

## Materials and Methods

### Plant material and pathogen

*Clavibater michiganense* subsp. *sepedonicum* (Spieckermann & Kotthoff) Davis et al. (ATCC 33113) was provided by the Chinese Academy of Agricultural Science. *Laminaria japonica* was purchased from a local market.

### Optimization of production of antimicrobial substances

#### Solvent selection

*L. japonica* was washed with distilled water [84]. The dried samples were finely ground and pulverized using a blender [85]. The powdered *L. japonica* (10 g of each) was extracted with 100 mL of methanol, ethanol, acetone, chloroform, ethyl acetate, butanol, benzene or petroleum ether for 8 h at their corresponding solvent boiling temperatures. After filtration, the organic solvent was evaporated under reduced pressure and temperature by a vacuum rotary evaporator. Then, each sample was individually dissolved in DMSO to give a final concentration of 1 mg/mL. The extracts were evaluated for their antimicrobial activities as described below.

Antimicrobial activity was determined by an agar diffusion method (ADM) based on radial diffusion [86]. Suspensions of the microorganism (10^8^ cfu/mL) were used for the studies. The medium contained 1 L distilled water, 20 g agar, 10 g peptone, 5 g beef extract, and 5 g sodium chloride. The agar surface was perforated with holes (10 mm in diameter), and a 200 μL volume of sample was pipetted into each hole. The DMSO was used as the control since it does not inhibit microorganism growth [87]. After the diffusion of the solution in each hole, the plates were inverted and incubated at 28°C for 24 h. Then, the antimicrobial activity of the inhibition zone (mm) was recorded. Each treatment was replicated four times.

#### Effects of extraction temperature, extraction time and solid to liquid ratio on antimicrobial substance production

Three important factors were considered when evaluating the antimicrobial activity of *L. japonica* against *C. michiganense* subsp. *sepedonicum*: extraction temperature (°C), extraction time (h) and solid to liquid ratio (g: mL). Each factor was divided into seven levels, and for each experiment, a total of 10 g of *L. japonica* sample was added to the corresponding volume of ethanol and extracted as described in Table 5. Four replicates were used for each extraction. Then, the antimicrobial activity was analyzed by ADM. Each treatment was replicated ten times. From the results of the antimicrobial activity, three reasonable levels of each factor were chosen for orthogonal experimental design.

**Table 5:** Factors and levels of orthogonal experiment.

Factors	Conditions	Levels						
		1	2	3	4	5	6	7
Temperature (°C)	Extraction time 8 h	40	50	60	70	80	90	98
	Solid to liquid ratio 1:15							
Extraction time (h)	Temperature 80°C	2	4	6	8	10	12	14
	Solid to liquid ratio 1:15							
Solid to liquid ratio (g: mL)	Extraction time 8 h	1:10	1:15	1:20	1:25	1:30	1:35	1:40
	Temperature 80°C							

#### Orthogonal experimental design

Based on the results of the extraction temperature, extraction time and solid to liquid ratio, three controlling factors at three levels, denoted 1, 2, and 3, were selected to investigate the optimal extraction conditions (Table 1). The impact of three factors was studied using L_27_(3^13^) orthogonal design, with consideration of the interactions between the factors [88], [89]. Twenty–seven experiments were performed. The complete design matrix of 27 experiments and the obtained results are shown in Table 2. For each experiment, a total of 10 g of *L. japonica* sample was added to the corresponding volume of ethanol and extracted as described in Table 2. Then, the antimicrobial activity of extracts from each sample was analyzed by ADM. Each treatment was replicated twelve times. ANOVA was performed to analyze the obtained data by using the SPSS software package (version 17.0).

### Major antimicrobial compound analysis

#### Solvent partition of *L. japonica* alcoholic extracts

The *L. japonica* alcoholic extracts were evaporated to dryness and then sterile water was added. Then the sample was extracted using petroleum ether, chloroform, ethyl acetate, or *n*–butyl alcohol as the extraction solvent (1∶1, v/v). The process was repeated five times [90].

#### Mobile phase selection

In order to select the mobile phase for silica gel column chromatography, TLC analysis was carried out. Petroleum ether partitions were spotted on TLC plates and were developed in the following solvent systems according to the solvent polarity (from low to high): benzene, chloroform/benzene (1∶1), chloroform, cyclohexane/ethyl acetate (8∶2), chloroform/acetone (95∶5), benzene/acetone (9∶1), benzene/ethyl acetate (8∶2), chloroform/diethyl ether (9∶1), petroleum ether/ethyl acetate (2∶1), benzene/diethyl ether (6∶4), chloroform/diethyl ether (8∶2), chloroform/methanol (99∶1), benzene/methanol (9∶1), chloroform/diethyl ether (6∶4), chloroform/methanol (95∶5), chloroform/acetone (7∶3), benzene/ethyl acetate (3∶7), ethyl acetate/methanol (99∶1), chloroform/methanol (9∶1) and methanol. The spots were visualized with iodine vapor. The solvent system that yielded the most spots was selected as the mobile phase for silica gel column chromatography.

#### Isolation of active substances from the petroleum ether phase with the best antimicrobial activity

The petroleum ether phase was purified by silica gel column chromatography. To obtain gradient elution, the mobile phase (petroleum ether and ethyl acetate (100∶0, 100∶1, 100∶2, 100∶3, 100∶5, 100∶7, 100∶10, 100∶15, 100∶20, 100∶30, 100∶50, 100∶100, 0:100, v/v)) was added to the column at 3–4 drops/s. A total of 294 fractions (bottles) were collected, 15 mL per bottle. The constituents in each fraction were compared by TLC. According to the TLC assay, all the fractions were amalgamated into nineteen groups. The nineteen groups were then evaporated to dryness. The dried fractionations of the nineteen groups were dissolved in DMSO to give a final concentration of 1 mg/mL for the antimicrobial activity assay (ADM). Each treatment was replicated five times.

#### Analysis of antimicrobial compounds using GC–MS

Based on the antimicrobial activity assay, the chemical constituents of the most efficacious group were determined using a GC–MS method [91]. One μL of diluted sample (1∶100 v/v, in acetone) was injected into the GC–MS (Thermo Trace 2000–Polaris Q, Thermo Finnigan Co., California, USA) operating in a splitless mode. The sample was separated on an Rtx–5MS column (length = 30 m, i.d. = 0.25 mm, thickness = 0.25 μm). The carrier gas was helium, and the flow rate was 1 mL/min. The column temperature was initially set at 80°C for 4 minutes, then gradually increased to 150°C at 15°C/min, and finally increased to 250°C at 10°C/min and held for 15 minutes. The MS total time was 30 minutes. The source and transfer line temperature were set at 250°C and 280°C, respectively. The mass spectrometer was operated in the 70 eV EI mode with scanning from 35 amu to 500 amu every 0.5 s. The major constituents were identified by the relative percent peak area of the TIC from the MS signal and identified by comparing their mass fragmentation pattern with those stored in the spectrometer database using NIST05.LIB (National Institute of Standards and Technology), and with data reported in the literature.

### Investigation of antimicrobial mechanism

#### Determination of MIC

The MIC of Fr.3 against *C*. *michiganense* subsp. *sepedonicum* was determined by the ADM method as described above. Fr.3 was first diluted to a concentration of 0.64 mg/mL, and then further diluted in DMSO to produce eight concentrations ranging from 0.64 mg/mL to 0.005 mg/mL. The MIC value was defined as the lowest concentration at which a visible inhibition zone around the hole was observed compared with the control (DMSO) [92], [93]. Each treatment was replicated four times.

#### Bactericidal kinetic assay

The bactericidal kinetic assay was performed by using appropriate concentrations of Fr.3, including 1/2MIC, MIC and 2MIC. *C*. *michiganense* subsp. *sepedonicum* (logarithmic phase) was adjusted to 10^6^ cfu/mL, and was then incubated with three concentrations of Fr.3 (1/2MIC, MIC and 2MIC). Samples for viable cell counting were taken every hour for 24 h. The solution without Fr.3 was used as a control [93]–[96].

#### SEM

Logarithmic phase cells of *C. michiganense* subsp. *sepedonicum* were treated with Fr.3 at MIC and 2MIC for 8 h. After incubation, the sample was first fixed with 4% gluteraldehyde at 4°C overnight. A second fixation procedure in 4% osmium tetraoxide (OSO_4_) was then carried out at 4°C for 2 h. The fixed samples were rinsed with phosphate buffer (PBS) for 10–15 minutes, and then were submerged in gradually increased ethanol concentrations (30%, 50%, 70%, 80%, 90%) for 20 minutes each, and finally submerged in 100% ethanol three times at 20 minutes intervals to ensure full dehydration. The samples were critical–point dried with CO_2_ in an HCP–2 critical-point dryer (Hitachi, Japan), and coated with IB–5 gold–palladium (Eiko, Japan). Samples were viewed at 25 kV accelerating voltage in a scanning electron microscope (JSM–35C, JEOL, Japan).

#### TEM

Logarithmic phase cells of *C. michiganense* subsp. *sepedonicum* were treated with Fr.3 at the MIC for 8 h. The samples were fixed in 3% glutaraldehyde followed by 4% OSO_4_. Then, the samples were dehydrated in gradually increasing acetone solutions, and embedded in Epon812. Ultrathin sections were cut and stained with uranyl acetate and lead citrate. The samples were examined using a JEM–1011(JEOL, Japan) transmission electron microscope operated at 80 kV.

#### Permeability of the cell wall and cell membrane

AKP was examined to determine the cell wall integrity of *C*. *michiganense* subsp. *sepedonicum*. Bacteria cells were incubated to logarithmic phase, and then mixed with different concentrations (1/5MIC, 1/2MIC, MIC and 2MIC) of Fr.3. The suspensions were incubated at 28°C for 2 h, 4 h, 6 h, 8 h and 10 h. Cells without Fr.3 were used as the control. At each time interval, the cells were centrifuged at 4000 rpm (1484×g) and the supernatant was examined using a kit purchased from Nanjing Jiancheng Bioengineering Institute (Nanjing, China). All the measurements were done in triplicate.

The cell membrane integrity of *C*. *michiganense* subsp. *sepedonicum* was examined by determination of the release of material by absorption at 260 nm [97]–[99]. Bacteria cells (logarithmic phase) were mixed with different concentrations (1/5MIC, 1/2MIC, MIC and 2MIC) of Fr.3. The suspensions were incubated at 28°C for 2 h, 4 h, 6 h, 8 h and 10 h. Cells without Fr.3 were used as the control. At each time interval, the cell was centrifuged at 4000 rpm (1484×g) and the absorbance of the obtained supernatant was measured at OD_260nm_ using a UV/VIS spectrophotometer (752pc, Shanhai Guangpu Instrument Co., LTD, China). All the measurements were done in triplicate.

The membrane potential of *C*. *michiganense* subsp. *sepedonicum* treated with Fr.3 was analyzed by rhodamine 123 staining followed by flow cytometry analysis [100]. Rhodamine 123 is a cell–permeable cationic fluorescent dye. *C*. *michiganense* subsp. *sepedonicum* (logarithmic phase) was mixed with different concentrations (1/5MIC, 1/2MIC, MIC and 2MIC) of Fr.3. The cells that were not treated with Fr.3 were used as the control. The suspensions were incubated at 28°C for 8 h. The cells were washed twice with PBS, and subsequently stained with 20 μM of rhodamine 123 in total darkness at room temperature for 30 minutes. The population of cells exhibiting green fluorescence was quantified using FACS Calibur flow cytometer (Becton Dickinson, United States), and the fluorescence micrographs were taken using a fluorescence microscope (Olympus microscope (BX51, Japan) digital microscope camera (DP72 Japan)) at an excitation wavelength of 507 nm and emission wavelength of 529 nm.

#### Determination of ROS, SOD, and CAT activities

The intracellular ROS was determined using the 2,7–dichlorofluorescin diacetate (DCFH–DA) assay. *C*. *michiganense* subsp. *sepedonicum* (logarithmic phase) was mixed with different concentrations (1/5MIC, 1/2MIC, MIC and 2MIC) of Fr.3. The cells that were not treated with Fr.3 were used as the control. The suspensions were incubated at 28°C for 8 h. Subsequently, cells were collected by centrifugation, and then the pellet was resuspended in DCFH–DA solution (20 μmol/L) in total darkness at room temperature for 30 minutes. Then these samples were washed to remove the excess DCFH–DA and adjusted to OD_630_ to 0.3 with PBS. The widths of both the excitation slit and the emission slit were set to 5 nm. Fluorescence intensity was measured at an excitation wavelength of 488 nm and an emission wavelength of 535 nm using a fluorescence spectrophotometer (F–280, Tianjin, Gangdong Sci & Tech Development Co., LTD, China) [101], [102].

*C. michiganense* subsp. *sepedonicum* was grown to logarithmic phase, and treated with Fr.3 at 1/5MIC, 1/2MIC, MIC, and 2MIC. Cells without Fr.3 treatment were used as the control. All samples were incubated at 28°C for 8 h. Then, 10 mL of each sample was centrifuged at 4000 rpm (1484×g) and was resuspended in 1 mL PBS. Each sample was sonicated using a probe sonicator at 200 W for 15minutes (working mode: 2 s pulse followed by a 4 s stop). The suspension was centrifuged at 12000 rpm (13363×g) for 15 minutes at 4°C, and then the SOD and CAT, in the supernatant were examined using a kit purchased from Nanjing Jiancheng Bioengineering Institute (Nanjing, China) [103].

#### Cytosolic Ca^2+^ concentration determination

To detect the cytosolic Ca*^2+^* levels, the Ca*^2+^* specific fluorescent dye, Fluo3–AM, was loaded into the cells, and the samples were analyzed by flow cytometry [104]. *C*. *michiganense* subsp. *sepedonicum* (logarithmic phase) was mixed with different concentrations (1/5MIC, 1/2MIC, and MIC) of Fr.3. The cells without Fr.3 treatment were used as the control. The suspensions were incubated at 28°C for 8 h. The cells were washed twice with PBS, and subsequently stained with 4 μM of Fluo3–AM in total darkness at room temperature for 60minutes. The population of cells exhibiting green fluorescence was quantified using FACS Calibur flow cytometer (Becton Dickinson, USA), and the fluorescence micrographs were taken using an excitation wavelength of 490 nm and emission wavelength of 520 nm.

#### Effect of Fr.3 on cellular respiration

*C*. *michiganense* subsp. *sepedonicum* (logarithmic phase) was mixed with different concentrations (1/2MIC, MIC, and 4MIC) of Fr.3. The cells without Fr.3 treatment were used as the control. Samples for dissolved oxygen (mg/L) testing were taken every hour for a 24 h period. Each treatment was replicated three times.

*C*. *michiganense* subsp. *sepedonicum* (logarithmic phase) was inoculated into liquid medium. The liquid medium was stirred for 5 minutes and then the initial dissolved oxygen (V_0_) was determined. The samples were mixed with propanedioic acid (1/5MIC), iodoacetic acid (1/5MIC), sodium phosphate (1/5MIC) or Fr.3 (1/5MIC) and the cells without any treatment were used as the control. After 8 h, the final dissolved oxygen of the treatment group (V_1_^'^) and the control group (V_0_^'^) were determined. The inhibition rate and overlay rate were evaluated as follows:
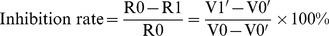



R_0_ =  The respiration rate of *C*. *michiganense* subsp. *sepedonicum* without treatment

R_1_ =  The respiration rate of *C*. *michiganense* subsp. *sepedonicum* induced by propanedioic acid (1/5MIC), iodoacetic acid (1/5MIC), sodium phosphate (1/5MIC) or Fr.3 (1/5MIC).
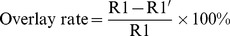



R_1_ =  The respiration rate of *C*. *michiganense* subsp. *sepedonicum* induced by Fr.3 (1/5MIC).

R_1_^'^ =  The respiration rate of *C*. *michiganense* subsp. *sepedonicum* induced by the combined action of Fr.3 (1/5MIC) and propanedioic acid (1/5MIC), the combined action of Fr.3 (1/5MIC) and iodoacetic acid (1/5MIC), or the combined action of Fr.3 (1/5MIC) and sodium phosphate (1/5MIC) [105].

*C. michiganense* subsp. *sepedonicum* was grown to logarithmic phase, and treated with Fr.3 at 1/5MIC, 1/2MIC, MIC, and 2MIC. Cells without Fr.3 were used as the control. All samples were incubated at 28°C for 8 h. A total of 10 mL of each sample was centrifuged at 4000 rpm (1484×g) and was resuspended in 1 mL sodium chloride (0.8%). Then each sample was sonicated using a probe sonicator at 200 W for 15 minutes (working mode: pulse 2 s followed by 4 s stop). The suspension was centrifuged at 12000 rpm (13363×g) for 15 minutes at 4°C, and then HK, PK, LDH, MDH, SDH and total ATP ase (T–ATPase) activities in the supernatant were examined using a kit purchased from Nanjing Jiancheng Bioengineering Institute (Nanjing, China) [106], [107]

#### SDS–PAGE

Logarithmic phase cells of *C. michiganense* subsp. *sepedonicum* were treated with Fr.3 at 1/2MIC, MIC, 2MIC and 4MIC for 8 h. Cells were collected by centrifugation and mixed with SDS sample buffer, incubated at 100°C for 5 minutes, and loaded onto 15% SDS-PAGE gels. Then, the gels were stained with Coomassie blue R-250 for 30 minutes and washed in destainer for 2 h [108].

#### Effect of Fr.3 on nucleic acids

The intracellular content of DNA and RNA were determined using DAPI staining assay. C. *michiganense* subsp. *sepedonicum* (logarithmic phase) was mixed with different concentrations (1/5MIC, 1/2MIC, MIC and 2MIC) of Fr.3. The cells without Fr.3 treatment was used as the control. The suspensions were incubated at 28°C for 8 h. Subsequently, the cells (2 mL) were incubated with 5 μL (0.1 mg/mL) DAPI in total darkness at room temperature for 30 minutes. Then these samples were washed to remove the excess DAPI and adjusted to an OD_630_ of 0.3 with PBS. The widths of both the excitation slit and the emission slit were set to 5 nm. Fluorescence intensity and fluorescence micrographs were determined using a fluorescence spectrophotometer (F–280, Tianjin, Gangdong Sci & Tech Development Co., LTD, China) and fluorescence microscope (Olympus microscope (BX51, Japan) equipped with a digital microscope camera (DP72 Japan)). The DNA in C. *michiganense* subsp. *sepedonicum* was measured at an excitation wavelength of 364 nm and emission wavelength of 450 nm. The RNA in C. *michiganense* subsp. *sepedonicum* was measured at an excitation wavelength of 400 nm and an emission wavelength of 465 nm [107].

Bacteria genomic DNA was extracted using bacterial genomic DNA isolation kit (Shangon Biotech (Shanghai) Co., LTD, China). The purity of the extracted genomic DNA was evaluated by the optical density ratio of 260 to 280 nm (OD260/OD280 = 1.80). DNA was mixed with increasing amounts of Fr.3 (final concentrations were 1/5MIC, 1/2MIC, MIC, and 2MIC) at room temperature for 2 h. DNA from cells without Fr.3 treatment was used as the control. After adding 10 μL of loading buffer, the migration of DNA was assessed by electrophoresis using a 0.8% agarose gel in 1×TAE buffer and stained using EB. Gel retardation was visualized under UV illumination using a gel imaging system (Shanhai Jiapeng Technology Co., LTD, China) [109].

Bacteria genomic DNA (OD_260_ = 0.5) was treated with Fr.3 at 1/5MIC, 1/2MIC, MIC, and 2MIC. DNA from cells without Fr.3 treatment was used as the control. After 5 h, the UV–visible spectra were measured at room temperature with a UV/VIS spectrophotometer (752pc, Shanhai Guangpu Instrument Co., LTD, China). Each sample solution was scanned from 220–380 nm.

Competitive binding assays were carried out as described by Li [110] with some modifications, using a F–280 fluorescence spectrophotometer (Tianjin Gangdong Sci & Tech Development Co., LTD, China). Bacteria genomic DNA was adjusted to an OD_260_ of 0.5. Then these samples were treated with Fr.3 at 1/2MIC, MIC, and 2MIC. DNA from cells without Fr.3 treatment was used as the control. After 5 h, 15 μL EB (0.1 mg/mL) was added to the DNA solution (2 mL). The EB–DNA mixture was incubated in the dark and the fluorescence spectra were measured for each test solution after 15 minutes. The solutions were excited at 493 nm, and the spectra were recorded from 500 to 700 nm. The widths of both the excitation slit and the emission slit were set to 2 nm.

Cell cycle analysis was performed by staining DNA with propidium iodide (PI, Sigma–Aldrich). Cells were collected after being cultured with Fr.3 at 1/5MIC, 1/2MIC, and MIC concentrations for 8 h. The cells without Fr.3 treatment were used as the control. Cell cycle analysis was performed by fixing cells with 75% alcohol in PBS. Cells were collected by centrifugation, and then the pellet was resuspended in PI solution (50 μg/mL). Flow cytometric analysis was done with a FACS Calibur flow cytometer (Becton Dickinson) and the data were analyzed by Cellquest software [111].

### Statistical analysis

One–way analysis of variance (ANOVA) was performed on the data using the SPSS statistic program (Version 17.0). The significance was determined by means of Duncan’s Multiple Range Test (*P*<0.05).

## References

[1] Navarro C, Abelenda JA, Cruz–Oró E, Cuéllar CA, Tamaki S, et al (2011) Control of flowering and storage organ formation in potato by FLOWERING LOCUS T. Nature 478: 119–122. 21947007 10.1038/nature10431

[2] van der Wolf JM, van Beckhoven JRCM, Hukkanen A, Karjalainen R, Müller P (2005) Fate of *Clavibacter michiganensis* ssp. *sepedonicus*, the causal organism of bacterial ring rot of potato, in weeds and field crops. J Phytopathol 153(6): 358–365.

[3] van Beckhoven JRCM, Stead DE, van der Wolf JM (2002) Detection of *Clavibacter michiganensis* subsp. *sepedonicus* by AmpliDet RNA, a new technology based on real time monitoring of NASBA amplicons with a molecular beacon. J Appl Microbiol 93(5): 840–849. 12392531 10.1046/j.1365-2672.2002.01765.x

[4] Qiu YE (2004) The occurrence and control of ring–rot in potato. Xinjiang Agric Sci (41): 88–89.

[5] Chen Y, Yue XL, Wang YC (2010) Features and integrated management of ring–rot in potato. J Shanxi Agric Sci 38(7): 140–141.

[6] Secor GA, de Buhr L, Gudmestad NC (1987) Chemical sanitation for bacterial ring rot control. Am Potato J 64: 699–700.

[7] Knight SC, Anthony VM, Brady AM, Greenland AJ, Heaney SP, et al (1997) Rationale and perspectives on the development of fungicides. Annu Rev Phytopathol 35: 349–372. 15012528 10.1146/annurev.phyto.35.1.349

[8] Cardoso RA, Pires LTA, Zucchi TD, Zucchi FD, Zucchi TMAD (2010) Mitotic crossing–over induced by two commercial herbicides in diploid strains of the fungus *Aspergillus nidulans* . Gen Mol Res 9: 231–238. 10.4238/vol9-1gmr688 20198578

[9] Ippolito A, Nigro F (2000) Impact of preharvest application of biological control agents on postharvest diseases of fresh fruits and vegetables. Crop Prot 19(8–10): 715–723.

[10] Brady NC (1984) The nature and properties of soils. Mac Millan Publishing Company, New York, p. 528.

[11] Asano T, Miwa A, Maeda K, Kimura M, Nishiuchi T (2013) The secreted antifungal protein thionin 2.4 in *Arabidopsis thaliana* suppresses the toxicity of a fungal fruit body lectin from *Fusarium graminearum* . Plos Pathog 9(8): e1003581. 23990790 10.1371/journal.ppat.1003581 PMC3749967

[12] Chen Y, Dai G (2012) Antifungal activity of plant extracts against *Colletotrichum lagenarium*, the causal agent of anthracnose in cucumber. J Sci Food Agric 92(9): 1937–1943. 22246784 10.1002/jsfa.5565

[13] Castro MS, Fontes W (2005) Plant defense and antimicrobial peptides. Protein Peptide Lett 12(1): 11–16. 15638798

[14] Rafiqi M, Ellis JG, Ludowici VA, Hardham AR, Dodds PN (2012) Challenges and progress towards understanding the role of effectors in plant–fungal interactions. Curr Opin Plant Biol 15: 477–482. 22658704 10.1016/j.pbi.2012.05.003

[15] Ahuja I, Kissen R, Bones AM (2012) Phytoalexins in defense against pathogens. Trends Plant Sci 17(2): 73–90. 22209038 10.1016/j.tplants.2011.11.002

[16] Peng Z, Liu M, Fang Z, Wu J, Zhang Q (2012) Composition and cytotoxicity of a novel polysaccharide from brown alga (*Laminaria japonica*). Carbohyd Polym 89(4): 1022–1026. 10.1016/j.carbpol.2012.03.043 24750908

[17] Li N, Zhang Q, Song J (2005) Toxicological evaluation of fucoidan extracted from *Laminaria japonica* in Wistar rats. Food Chem Toxicol 43(3): 421–426. 15680677 10.1016/j.fct.2004.12.001

[18] Tseng ZCCK, Chang JCF (1984) Chinese seaweeds in herbal medicine. Hydrobiologia 22: 152–154.

[19] Go H, Hwang HJ, Nam TJ (2010) A glycoprotein from *Laminaria japonica* induces apoptosis in HT-29 colon cancer cells. Toxicol *in vitro* 24(6): 1546–1553. 20615460 10.1016/j.tiv.2010.06.018

[20] Tseng CK (2001) Algal biotechnology industries and research activities in China. J Appl Phycol 13: 375–380.

[21] Wu P, Imlay JA, Shang JK (2010) Mechanism of *Escherichia coli* inactivation on palladium–modified nitrogen–doped titanium dioxide. Biomaterials 31(29): 7526–7533. 20637502 10.1016/j.biomaterials.2010.06.032 PMC3051420

[22] Schepers E, Glorieux G, Dhondt A, Leybaert L, Vanholder R (2009) Flow cytometric calcium flux assay: Evaluation of cytoplasmic calcium kinetics in whole blood leukocytes. J Immunol Methods 348(1–2): 74–82. 19616551 10.1016/j.jim.2009.07.002

[23] Lepecq JB, Paoletti C (1967) A fluorescent complex between ethidium bromide and nucleic acid: Physical-chemical characterization. J Mol Biol 27(1): 87–106. 6033613 10.1016/0022-2836(67)90353-1

[24] Geng F, Wang W, Zhou T (2011) Antibacterial mechanisms of Fructus mume extract against *Listeria innocua* . Food Sci 32: 88–93.

[25] Xiong JH, Liu ZH, Wang KQ (2007) Extraction and purification of polyphenols in Ilex paraguarensis and its antibacteriostatic activities. Food Mach 23(5): 78–80.

[26] Liu RM, Zhang K, Cui QX (2002) Study on extraction of pumpkin seed oil by supercritical CO_2_ . Food Ferm Indus 29: 61–65.

[27] Yang DM, Zhu XY, Feng LD, Bi Y, Ying TJ (2010) Study on the extraction technology of the antibacterial components from *Potentilla ansterina* L. J Chin Inst Food Sci Technol 10: 47–51.

[28] Bernardo–Gil MG, Casquiho M, Esquível MM, Ribeiro MA (2009) Supercritical fluid extraction of fig leaf gourd seeds oil: fatty acids composition and extraction kinetics. J Supercrit Fluids 49(1): 32–36.

[29] Sun L, Lee HK (2003) Optimization of microwave–assisted extraction and supercritical fluid extraction of carbamate pesticides in soil by experimental design methodology. J Chromatogr A 1014(1–2): 165–177. 14558622 10.1016/s0021-9673(03)00574-0

[30] Antony J (2006) Taguchi or classical design of experiments: a perspective from a practitioner. Sens Rev 26(3): 227–230.

[31] Nishida M, Yashiki M, Namera A, Kimura K (2006) Single hair analysis of methamphetamine and amphetamine by solid phase microextraction coupled with in matrix derivatization. J Chromatogr B 842(2): 106–110. 10.1016/j.jchromb.2006.07.039 16971192

[32] Kilickap E (2010) Modeling and optimization of burr height in drilling of Al–7075 using Taguchi method and response surface methodology. Int J Adv Manuf Technol 49(9–12): 911–923.

[33] Díez C, Barrado E, Marinero P, Sanz M (2008) Orthogonal array optimization of a multiresidue method for cereal herbicides in soils. J Chromatogr A 1180(1–2): 10–23. 18184615 10.1016/j.chroma.2007.12.036

[34] Zhen Y, Jiang HX, Lin XP (2004) Chemical analysis of lipid compound and its antibacterial and antifungal activities in *Pachydictyon coriaceum* . Marine Sci 28(10): 42–44.

[35] Karabay-Yavasoglu NU, Sukatar A, Ozdemir G, Horzum Z (2007) Antimicrobial activity of volatile components and various extracts of the red alga *Jania rubens* . Phytotherapy Res 21(2): 153–156. 10.1002/ptr.2045 17128433

[36] Lupea AX, Radu F, Gergen I (2003) Abietic acid ammonium salts, biodegradable surface-active agents with antimicrobial activity. Revista De Chimie 54(11): 923–926.

[37] Huang CB, George B, Ebersole JL (2010) Antimicrobial activity of n–6, n–7 and n–9 fatty acids and their esters for oral microorganisms. Arch Oral Biol 55(8): 555–560. 20541177 10.1016/j.archoralbio.2010.05.009 PMC2902640

[38] Dhankhar S, Dhankhar S, Kumar M, Ruhil S, Balhara M, et al (2012) Analysis toward innovative herbal antibacterial & antifungal drugs. Recent Pat Antiinfect Drug Discov 7: 242–248. 23072646 10.2174/157489112803521931

[39] Kumar A, Jayachandran T, Aravindhan P, Deecaraman D, Ilavarasan R, et al (2009) Neutral components in the leaves and seeds of *Syzygium cumini* . Afr J Pharm Pharmaco 3(11): 560–561.

[40] Sukatar A, Karabay–Yavaşoglu NU, Ozdemir G, Horzum Z (2006) Antimicrobial activity of volatile component and various extracts of *Enteromorpha linza* (Linnaeus) J. Agardh from the coast of Izmir, Turkey. Ann Microbiol 56(3): 275–279.

[41] Ozdemir G, Horzum Z, Sukatar A, Karabay–Yavasoglu NU (2006) Antimicrobial activities of volatile components and various extracts of *Dictyopteris membranaceae*. and *Cystoseira barbata*. from the coast of Izmir, Turkey. Pharm Biol 44(3): 183–188.

[42] Palic R, Stojanovic G, Alagic S, Nikolic M, Lepojevic Z (2002) Chemical composition and antimicrobial activity of the essential oil and CO_2_ extracts of the oriental tobacco, Prilep. Flavour Frag J 17(5): 323–326.

[43] Yayli N, GüleS˛ C, Üc˛üncü O, YaŞar A, Ülker S, et al (2006) Composition and antimicrobial activities of volatile components of *Minuartia meyeri* . Turk J Chem 30: 71–76.

[44] Bagheri–Gavkosh S, Bigdeli M, Shams–Ghahfarokhi M, Razzaghi–Abyaneh M (2009) Inhibitory effects of *Ephedra major* host on *Aspergillus parasiticus* growth and aflatoxin production. Mycopathologia 168(5): 249–255. 19557546 10.1007/s11046-009-9220-x

[45] Rizwan K, Zubair M, Rasool N, Riaz M, Zia–Ul–Haq M, et al (2012) Phytochemical and biological studies of *Agave attenuate* . Int J Mol Sci 13: 6440–6451. 22754375 10.3390/ijms13056440 PMC3382814

[46] Sinek K, Iskender NY, Yayli B, Karaoglu SA, Yayli N (2012) Antimicrobial Activity and chemical composition of the essential oil from *Campanula glomerata* L. subsp *hispida* (Witasek) Hayek. Asian J Chem 24: 1931–1934.

[47] Boussaada O, Ammar S, Mahjoub MA, Saidana D, Chriaa J, et al (2009) Chemical composition and antimicrobial activity of volatile components from capitula, stems–leaves and aerial parts of *Mantisalca duriaei* Briq. et Cavill growing wild in Tunisia. J Essent Oil Res 21(2): 179–184.

[48] Gulec C, Yayli N, Yesilgil P, Terzioglu S, Yayli N (2007) Activities of the essential oil from the flowers of delphinium formosum. Asian J Chem 19: 4069–4074.

[49] Passos XS, Castro ACM, Pires JS, Garcia ACF, Campos FC, et al (2003) Composition and antifungal activity of the essential oils of *Caryocar brasiliensis* . Pharm Biol 41: 319–324.

[50] Zito P, Sajeva M, Bruno M, Maggio A, Rosselli S, et al (2011) Essential oil composition of the fruits of *Periploca laevigata* Aiton subsp. *angustifolia* (Labill.) Markgraf (Apocynaceae – Periplocoideae). Nat Prod Res 25(14): 1339–1346. 21859258 10.1080/14786419.2010.535157

[51] Valiei M, Shafaghat A, Salimi F (2011) Chemical composition and antimicrobial activity of the flower and root hexane extracts of *Althaea officinalis* in Northwest Iran. J Med Plants Res 5(32): 6972–6976.

[52] Wang GL, Yue J, Li HY, Fang HJ (2005) Extraction of anthocyanin from sweetpotato by macroporous resin and its bacteriostatic mechanism. Sci Agr Sinica 38(11): 2321–2326.

[53] Ultee A, Bennik MHJ, Moezelaar R (2002) The phenolic hydroxyl group of carvacrol is essential for action against the food–borne pathogen *Bacillus cereus* . Appl Environ Microb 68(4): 1561–1568. 10.1128/AEM.68.4.1561-1568.2002 PMC123826 11916669

[54] Sikkema J, de Bont JA, Poolman B (1995) Mechanisms of membrane toxicity of hydrocarbons. Microbiol Mol Biol 59(2): 201–222. 10.1128/mr.59.2.201-222.1995 PMC239360 7603409

[55] Ibrahim HR, Higashiguchi S, Koketsu M, Juneja LR, Kim M, et al (1996) Partially unfolded lysozyme at neutral pH agglutinates and kills Gram–negative and Gram–positive bacteria through membrane damage mechanism. J Agri Food Chem 44(12): 3799–3806.

[56] Holley RA, Patel D (2005) Improvement in shelf–life and safety of perishable foods by plant essential oils and smoke antimicrobials. Food Microbiol 22: 273–292.

[57] Khan MS, Ahmad I (2011) *In vitro* antifungal, anti–elastase and anti–keratinase activity of essential oils of *Cinnamomum*–, *Syzygium*– and *Cymbopogon*–species against *Aspergillus fumigatus* and *Trichophyton rubrum* . Phytomedicine 19(1): 48–55. 21893402 10.1016/j.phymed.2011.07.005

[58] Hara S, Yamakawa M (1995) Moricin, a novel type of antibacterial peptide isolated from the silkworm, *Bombyx mori* . J Biol Chem 270: 29923–29927. 8530391 10.1074/jbc.270.50.29923

[59] Cheng KJ, Ingram JM, Costerton JW (1970) Release of alkaline phosphatase from Cells of *Pseudomonas aeruginosa* by manipulation of cation concentration and of pH. J Bacteriol 104(2): 748–753. 4992367 10.1128/jb.104.2.748-753.1970 PMC285053

[60] Kohanski MA, Dwyer DJ, Hayete B, Lawrence CA, Collins JJ (2007) A common mechanism of cellular death induced by bactericidal antibiotics. Cell 130(5): 797–810. 17803904 10.1016/j.cell.2007.06.049

[61] Yang W, Tang Z, Zhou F, Zhang W, Song L (2013) Toxicity studies of tetracycline on *Microcystis aeruginosa* and *Selenastrum capricornutum* . Environ Toxicol Pharmacol 35(2): 320–324. 23380052 10.1016/j.etap.2013.01.006

[62] Anastasiou D, Poulogiannis G, Asara JM, Boxer MB, Jiang J, et al (2011) Inhibition of pyruvate kinase M2 by reactive oxygen species contributes to cellular antioxidant responses. Science 334: 1278–1283. 22052977 10.1126/science.1211485 PMC3471535

[63] Bowler C, Montagu MV, Inze D (1992) Superoxide dismutase and stress tolerance. Annu Rev Plant Physiol Plant Mol Biol 43: 83–116.

[64] Halliwell B, Gutteridge JM (1984) Oxygen toxicity, oxygen radicals, transition metals and disease. Biochem J 219: 1–14. 6326753 10.1042/bj2190001 PMC1153442

[65] Halliwell B (1994) Free radicals and antioxidants: a personal view. Nutr Rev 52: 253–265. 7970288 10.1111/j.1753-4887.1994.tb01453.x

[66] Li Q, Peng S, Sheng Z, Wang Y (2010) Ofloxacin induces oxidative damage to joint chondrocytes of juvenile rabbits: Excessive production of reactive oxygen species, lipid peroxidation and DNA damage. Eur J Pharmacol 626(2–3): 146–153. 19818344 10.1016/j.ejphar.2009.09.044

[67] Foreman J, Demidchik V, Bothwell JH, Mylona P, Miedema H, et al (2003) Reactive oxygen species produced by NADPH oxidase regulate plant cell growth. Nature 422: 442–446. 12660786 10.1038/nature01485

[68] Mori IC, Schroeder JI (2004) Reactive oxygen species activation of plant Ca^2+^ channels. A signaling mechanism in polar growth, hormone transduction, stress signaling, and hypothetically mechanotransduction. Plant Physiol 135(2): 702–708. 15208417 10.1104/pp.104.042069 PMC514107

[69] Chen Y, Huang K, Gao JH, Ning ZX (1994) Inhibition of the respiration of microorganism by α–Bromo cinnamaldehyde an alkenoic acid esters. Food Fermentation Ind 3: 26–29.

[70] Yun B, Zhou L, Xie KP, Wang YJ, Xie MJ (2012) Antibacterial activity and mechanism of baicalein. Acta Pharm Sin 47(12): 1587–1592. 23460962

[71] Munger J, Bajad SU, Coller HA, Shenk T, Rabinowitz JD (2006) Dynamics of the cellular metabolome during human cytomegalovirus infection. Plos Pathog 2(12): e0020132. 10.1371/journal.ppat.0020132 PMC1698944 17173481

[72] Mattevi A, Bolognesi M, Valentini G (1996) The allosteric regulation of pyruvate kinase. FEBS Lett 389(1): 15–19. 8682196 10.1016/0014-5793(96)00462-0

[73] Rigden DJ, Phillips SEV, Michels PAM, Fothergill–Gilmore LA (1999) The structure of pyruvate kinase from Leishmania mexicana reveals details of the allosteric transition and unusual effector specificity. J Mol Biol 291(3): 615–635. 10448041 10.1006/jmbi.1999.2918

[74] Alteri CJ, Smith SN, Mobley HLT (2009) Fitness of *Escherichia coli* during urinary tract infection requires gluconeogenesis and the TCA cycle. Plos Pathog 5(5): e1000448. 19478872 10.1371/journal.ppat.1000448 PMC2680622

[75] Yao X, Zhu X, Pan S, Fang Y, Jiang F, et al (2012) Antimicrobial activity of nobiletin and tangeretin against *Pseudomonas* . Food Chem 132(4): 1883–1890.

[76] Lu X, Shen J, Jin X, Ma Y, Huang Y, et al (2012) Bactericidal activity of *Musca domestica* cecropin (Mdc) on multidrug–resistant clinical isolate of *Escherichia coli* . Appl Microbiol Biotechnol 95(4): 939–945. 22202966 10.1007/s00253-011-3793-2

[77] Han G, Yang P (2002) Synthesis and characterization of water–insoluble and water–soluble dibutyltin (IV) porphinate complexes based on the tris (pyridinyl) porphyrin moiety, their anti–tumor activity in vitro and interaction with DNA. J Inorg Biochem 91: 230–236. 12121780 10.1016/s0162-0134(02)00369-0

[78] Tarushi A, Psomas G, Raptopoulou CP, Kessissoglou DP (2009) Zinc complexes of the antibacterial drug oxolinic acid: structure and DNA–binding properties. J Inorg Biochem 103: 898–905. 19395041 10.1016/j.jinorgbio.2009.03.007

[79] Senthil Kumar R, Sasikala K, Arunachalam S (2008) DNA interaction of some polymer–copper (II) complexes containing 2,2'–bipyridyl ligand and their antimicrobial activities. J Inorg Biochem 102(2): 234–241. 17920685 10.1016/j.jinorgbio.2007.08.005

[80] Efthimiadou EK, Karaliota A, Psomas G (2008) Structure, antimicrobial activity and DNA–binding properties of the cobalt (II) – sparfloxacin complex. Bioorg Med Chem lett 18(14): 4033–4037. 18579377 10.1016/j.bmcl.2008.05.115

[81] Efthimiadou EK, Karaliota A, Psomas G (2010) Metal complexes of the third–generation quinolone antimicrobial drug sparfloxacin: Structure and biological evaluation. J Inorg Biochem 104(4): 455–466. 20106531 10.1016/j.jinorgbio.2009.12.019

[82] Chen JC, Stephens C (2007) Bacterial cell cycle: completing the circuit. Curr Biol 17(6): 203–206. 17371757 10.1016/j.cub.2007.01.031

[83] Lieberthal W, Menza SA, Levine JS (1998) Graded ATP depletion can cause necrosis or apoptosis of cultured mouse proximal tubular cells. Am J Physiol Ren Physiol 274: 315–327. 10.1152/ajprenal.1998.274.2.F315 9486226

[84] Abdel–Monaim MF, Abo–Elyousr KAM, Morsy KM (2011) Effectiveness of plant extracts on suppression of damping–off and wilt diseases of lupine (*Lupinus termis Forsik*). Crop Prot 30(2): 185–191.

[85] Choi YS, Choi JH, Han DJ, Kim HY, Kim HW, et al (2012) Effects of *Laminaria japonica* on the physico–chemical and sensory characteristics of reduced–fat pork patties. Meat Sci 91(1): 1–7. 22227100 10.1016/j.meatsci.2011.11.011

[86] Michielin EMZ, Salvador AA, Riehl CAS, Smânia Jr A, Smânia EFA, et al (2009) Chemical composition and antibacterial activity of *Cordia verbenacea* extracts obtained by different methods. Bioresource Technol 100(24): 6615–6623. 10.1016/j.biortech.2009.07.061 19683436

[87] Smânia Jr A, Delle Monache F, Smânia EFA, Cuneo RS (1999) Antibacterial activity of steroidal compounds isolated from *Ganoderma applanatum* (Pers.) Pat. (Aphyllophoromycetideae) fruit body. Int J Med Mushrooms 1(4): 325–330.

[88] Jia XY, Li NB, Luo HQ (2010) Determination of ursolic acid in force loquat capsule by ultrasonic extraction and ionic liquid based reverse dispersive LLME. Chromatographia 71: 839–843.

[89] Zhou W, Zhang X, Xie M, Chen Y, Li Y, et al (2010) Infrared–assisted extraction of adenosine from radix isatidis using orthogonal experimental design and LC. Chromatographia 72: 719–724.

[90] Wang HK, Yan YH, Wang JM, Zhang HP, Qi W (2012) Production and characterization of antifungal compounds produced by *Lactobacillus plantarum* IMAU10014. Plos One 7(1): e0029452. 10.1371/journal.pone.0029452 PMC3261852 22276116

[91] Muthukumar A, Eswaran A, Nakkeeran S, Sangeetha G (2010) Efficacy of plant extracts and biocontrol agents against *Pythium aphanidermatum* inciting chilli damping–off. Crop Prot 29(12): 1483–1488.

[92] Barros L, Calhelha RC, Vaz JA, Ferreira ICFR, Baptista P, et al (2007) Antimicrobial activity and bioactive compounds of Portuguese wild edible mushrooms methanolic extracts. Eur Food Res Technol 225(2): 151–156.

[93] Talibi I, Askarne L, Boubaker H, Boudyach EH, Msanda F, et al (2012) Antifungal activity of some Moroccan plants against *Geotrichum candidum*, the causal agent of postharvest citrus sour rot. Crop Prot 35: 41–46.

[94] Bakken LR, Olsen RA (1987) The relationship between cell size and viability of soil bacteria. Microb Ecol 13(2): 103–114. 24213209 10.1007/BF02011247

[95] Liu X, Zu Y, Fu Y, Yao L, Gu C, et al (2009) Antimicrobial activity and cytotoxicity towards cancer cells of *Melaleuca alternifolia* (tea tree) oil. Eur Food Res Technol 229(2): 247–253.

[96] Avila JG, de Liverant JG, Martınez A, Martĺneza G, Muñoza JL, et al (1999) Mode of action of *Buddleja cordata* verbascoside against *Staphylococcus aureu*s. J Ethnopharmacol 66(1): 75–78. 10432210 10.1016/s0378-8741(98)00203-7

[97] Chen CZ, Cooper SL (2002) Interactions between dendrimer biocides and bacterial membranes. Biomaterials 23(16): 3359–3368. 12099278 10.1016/s0142-9612(02)00036-4

[98] Politoff AL, Socolar SJ, Loewenstein WR (1969) Permeability of a cell membrane junction. J Gen Physiol 53(4): 498–515. 5778320 10.1085/jgp.53.4.498 PMC2202868

[99] Devi PK, Nisha SA, Sakthivel R, Pandian SK (2010) Eugenol (an essential oil of clove) acts as an antibacterial agent against *Salmonella typhi* by disrupting the cellular membrane. J Ethnopharmacol 130(1): 107–115. 20435121 10.1016/j.jep.2010.04.025

[100] Haines LR, Thomas JM, Jackson AM, Eyford BA, Razavi M, et al (2009) Killing of trypanosomatid parasites by a modified bovine host defense peptide, BMAP–18. Plos Neglect Trop Dis 3(2): e0000373. 10.1371/journal.pntd.0000373 PMC2628741 19190729

[101] Wang J, Xia XM, Wang HY, Lia PP, Wang KY (2013) Inhibitory effect of lactoferrin against gray mould on tomato plants caused by *Botrytis cinerea* and possible mechanisms of action. Int J Food Microbiol 161: 151–157. 23333340 10.1016/j.ijfoodmicro.2012.11.025

[102] Cheng X, Zhang W, Ji Y, Meng J, Guo H, et al (2013) Revealing silver cytotoxicity using Au nanorods/Ag shell nanostructures: disrupting cell membrane and causing apoptosis through oxidative damage. RSC Adv 3(7): 2296–2305.

[103] Shen HM, Shi CY, Shen Y, Ong CN (1996) Detection of elevated reactive oxygen species level in cultured rat hepatocytes treated with aflatoxin B1. Free Rad Bio Med 21(2): 139–146. 8818628 10.1016/0891-5849(96)00019-6

[104] Wang C, Zhou Y, Li S, Li HB, Tian LL, et al (2013) Anticancer mechanisms of temporin–1CEa, an amphipathic α–helical antimicrobial peptide, in Bcap–37 human breast cancer cells. Life Sci 92(20–21): 1004–1014. 23583573 10.1016/j.lfs.2013.03.016

[105] Song FP, Sheng ST, Hu TL, Wei JJ, Cao KQ (2009) Preliminary Studies on the mode of action of mangifer in against *Phytoph thora* infestans. Chin J Pest Sci 11(2): 213–218.

[106] Bradford MM (1976) A rapid and sensitive method for the guantitation of microgram quantities of protein utilizing the principle of protein–dye binding. Anal Biochem 72(1–2): 248–254. 942051 10.1016/0003-2697(76)90527-3

[107] Wang Q, Wang H, Xie M (2010) Antibacterial mechanism of soybean isoflavone on *Staphylococcus aureus* . Arch Microbiol 192(11): 893–898. 20734190 10.1007/s00203-010-0617-1

[108] Friedlander ML, Hedley DW, Taylor IW (1984) Clinical and biological significance of aneuploidy in human tumours. J Clin Pathol 37: 961–974. 6381555 10.1136/jcp.37.9.961 PMC498910

[109] Imura Y, Nishida M, Matsuzaki K (2007) Action mechanism of PEGylated magainin 2 analogue peptide. Biochim Biophys Acta Biom 1768(10): 2578–2585. 10.1016/j.bbamem.2007.06.013 17662233

[110] Li L, Shi Y H, Cheserek M J, Su G F, Le G W (2013) Antibacterial activity and dual mechanisms of peptide analog derived from cell-penetrating peptide against *Salmonella typhimurium* and *Streptococcus pyogenes* . Appl Microbiol Biotechnol 97(4): 1711–1723. 22923068 10.1007/s00253-012-4352-1

[111] Muirhead KA, Horan PK, Poste G (1985) Flow cytometry: present and future. Nat Biotechnol 3: 337.

